# Intradiol ring cleavage dioxygenases from herbivorous spider mites as a new detoxification enzyme family in animals

**DOI:** 10.1186/s12915-022-01323-1

**Published:** 2022-06-04

**Authors:** Christine Njiru, Wenxin Xue, Sander De Rouck, Juan M. Alba, Merijn R. Kant, Maksymilian Chruszcz, Bartel Vanholme, Wannes Dermauw, Nicky Wybouw, Thomas Van Leeuwen

**Affiliations:** 1 Laboratory of Agrozoology, Department of Plants and Crops, Faculty of Bioscience Engineering, Ghent University, Ghent, Belgium; 2 Department of Evolutionary and Population Biology, Institute for Biodiversity and Ecosystem Dynamics, University of Amsterdam, Amsterdam, The Netherlands; 3 Department of Chemistry and Biochemistry, University of South Carolina, Columbia, SC 29208 USA; 4 Department of Plant Biotechnology and Bioinformatics, Ghent University, Ghent, Belgium; 5 VIB Center for Plant Systems Biology, Ghent, Belgium; 6 Flanders Research Institute for Agriculture, Fisheries and Food (ILVO), Plant Sciences Unit, Merelbeke, Belgium; 7 Terrestrial Ecology Unit, Department of Biology, Faculty of Sciences, Ghent University, Ghent, Belgium

**Keywords:** Tetranychidae, Plant-herbivore interactions, Dioxygenases, Ortho-cleavage, Aromatic compounds

## Abstract

**Background:**

Generalist herbivores such as the two-spotted spider mite *Tetranychus urticae* thrive on a wide variety of plants and can rapidly adapt to novel hosts. What traits enable polyphagous herbivores to cope with the diversity of secondary metabolites in their variable plant diet is unclear. Genome sequencing of *T. urticae* revealed the presence of 17 genes that code for secreted proteins with strong homology to “intradiol ring cleavage dioxygenases (DOGs)” from bacteria and fungi, and phylogenetic analyses show that they have been acquired by horizontal gene transfer from fungi. In bacteria and fungi, DOGs have been well characterized and cleave aromatic rings in catecholic compounds between adjacent hydroxyl groups. Such compounds are found in high amounts in solanaceous plants like tomato, where they protect against herbivory. To better understand the role of this gene family in spider mites, we used a multi-disciplinary approach to functionally characterize the various *T. urticae DOG* genes.

**Results:**

We confirmed that *DOG* genes were present in the *T. urticae* genome and performed a phylogenetic reconstruction using transcriptomic and genomic data to advance our understanding of the evolutionary history of spider mite *DOG* genes. We found that *DOG* expression differed between mites from different plant hosts and was induced in response to jasmonic acid defense signaling. In consonance with a presumed role in detoxification, expression was localized in the mite’s gut region. Silencing selected *DOGs* expression by dsRNA injection reduced the mites’ survival rate on tomato, further supporting a role in mitigating the plant defense response. Recombinant purified DOGs displayed a broad substrate promiscuity, cleaving a surprisingly wide array of aromatic plant metabolites, greatly exceeding the metabolic capacity of previously characterized microbial DOGs.

**Conclusion:**

Our findings suggest that the laterally acquired spider mite DOGs function as detoxification enzymes in the gut, disarming plant metabolites before they reach toxic levels. We provide experimental evidence to support the hypothesis that this proliferated gene family in *T. urticae* is causally linked to its ability to feed on an extremely wide range of host plants.

**Supplementary Information:**

The online version contains supplementary material available at 10.1186/s12915-022-01323-1.

## Background

Plants produce a diverse set of aromatic compounds, ranging from aromatic amino acids to secondary metabolites that often function as defense compounds against herbivores and pathogens (e.g., flavonoids, terpenes and phenolics) or as building blocks for structural polymers (e.g., lignin and suberin) [1–4]. In addition, human activities have led to persistent and toxic man-made aromatic pollutants such as benzene, nitrophenols, organophosphates, chlorinated phenols, and hydrocarbons [5, 6]. Some species of bacteria and fungi are able to degrade these often complex organic and synthetic aromatic compounds and thus are important players in the global carbon cycle [5].

Microbial degradation of aromatic compounds involves a series of endogenous funneling reactions that convert a wide variety of organic compounds to either 3,4-dihydroxybenzoate (protocatechuate) or 1,2-dihydroxybenzene (catechol), simple monocyclic aromatic compounds (Figure S1). Both compounds are catabolized by the β-ketoadipate pathway (β-KAP), which first step is a ring cleavage reaction catalyzed by a class of non-heme iron-containing enzymes referred to as ring cleavage dioxygenases [6, 7]. Based on their cleavage mechanism, these enzymes are classified as either extradiol or intradiol ring cleavage dioxygenases.

The extradiol ring cleavage dioxygenases cleave protocatechuate and catechol rings adjacent to either of the hydroxyl groups (meta cleavage) to form semialdehydes [8, 9]. This class of enzymes is widespread across the tree of life. In addition, they typically exhibit large substrate promiscuity and catalyze multiple reactions in addition to the oxygen-mediated ring splitting, including epimerization, isomerization, and nucleophilic substitution [4, 5, 10–12].

Intradiol ring cleavage dioxygenases (hereafter abbreviated as DOGs) cleave protocatechuate and catechol rings between the two neighboring hydroxylated carbons (ortho-cleavage) to form dicarboxylic acid. Based on their substrate specificity, they are further classified as catechol 1,2-dioxygenases, protocatechuate 3,4-dioxygenases or hydroxyquinol 1,2-dioxygenases [4]. In contrast to extradiol ring cleavage dioxygenases, DOGs are believed to only catalyze oxygen-mediated ring cleavage reactions and are not as widespread across the tree of life. In the past, they were considered to be restricted to the genomes of bacteria and fungi. However, in 2011, Grbić et al. [13] reported the presence of a *DOG*-like family in the spider mite *Tetranychus urticae*, a polyphagous arthropod herbivore*.* Further studies showed that these *DOG* genes had been acquired by a single horizontal gene transfer (HGT) event from fungi and subsequently proliferated [13–15]. However, the timing and functional importance of the transfer event remains elusive. Schlachter et al. [16] uncovered that one paralog (*tetur07g02040*) codes for a DOG enzyme capable of cleaving the model substrates catechol and 4-methyl catechol in vitro*.* Additionally, this enzyme is active in a monomeric state, unlike bacterial and fungal DOGs [17–19].

The presence of a proliferated *DOG* gene family (with 17 paralogs in the London reference genome) in *T. urticae* suggests that these horizontally acquired genes functionally diversified and could contribute to the ability of *T. urticae* to metabolize the myriad of different aromatic compounds it encounters in its plant diet [20, 21]. Previous work supports this hypothesis by uncovering strong transcriptional responses of *DOG* genes upon short- and long-term transfers from kidney bean to other host plants that are defended by a diverse blend of aromatic compounds [14, 22, 23].

Studies on fungi and bacteria have reported that certain secreted DOGs exhibit the rare ability to cleave non-model complex substrates like procyanidins and catecholic intermediates of the lignin biosynthetic pathway such as caffeic acid and caffeoyl-CoA [19, 24]. In the current study, we functionally characterized the spider mite *DOGs* and investigated the hypothesis that they can cleave plant-derived aromatic compounds. We first examined the evolutionary histories of DOGs within the Tetranychoidea superfamily, including spider mites and false spider mites. We then assessed *DOG* transcript accumulation in different feeding and non-feeding mite developmental stages as well as in adults in response to various plant diets. We also established in which mite tissues *DOGs* are expressed via *in situ* hybridization. Furthermore, we assessed the effect of *DOG* knockdown via RNAi on spider mite performance on different host plants. We subsequently produced recombinant protein for 7 DOGs and tested to what extent they can cleave 33 aromatic plant compounds, many of which are known to be toxic to herbivores. Understanding the functional role of this unique enzyme family might not only help to understand why certain spider mite species like *T. urticae* thrive on so many host plants with markedly different chemical profiles but could also be a stimulus for the development of commercial applications that can efficiently degrade environmental pollutants.

## Materials and methods

### Mite strains and tomato cultivars

The London strain is an outbred reference laboratory *T. urticae* strain [13] and was maintained on potted bean plants (*Phaseolus vulgaris* L. cv “Prelude”). The AT-London strain is derived from London and is adapted to tomato [23]. AT-London was maintained on potted tomato plants (*Solanum lycopersicum* L. cv “Moneymaker”). The METI-resistant strain MR-VP was maintained on kidney bean leaves treated with foliar applications of a commercial formulation of tebufenpyrad [25]. Houten-1 was originally collected in a tomato greenhouse in Houten (The Netherlands) [26]. Santpoort-2 was collected from a spindle tree located in a natural park near Santpoort (The Netherlands) [27], whereas DeLier-1 was collected from *Ricinus communis* in a rural area close to De Lier (The Netherlands) [28]. Houten-1 mites were maintained on detached tomato leaves (cv “Castlemart” hereafter referred to as CM), whereas Santpoort-2 and Delier-1 were maintained on detached bean leaves (*P. vulgaris* L. cv “Speedy”). The Lahijan strain of *Panonychus citri* was originally collected in citrus orchards in Guilan (Iran) [29] and was maintained on detached sour orange leaves (*Citrus aurantium L.*). Mite strains were reared under laboratory conditions of 25 ± 1 °C, 60% relative humidity and a 16:8 h light to dark period. Here, we used tomato plants (CM) and the jasmonic acid mutant within a CM genetic background (hereafter referred to as *def-1*) in our experiments. Tomato plants were reared under greenhouse conditions (25:18 °C day to night, 50–60% relative humidity and 16:8 h light to dark photoperiod). Experiments involving potted tomato plants were carried out in a climate room (25 °C, 60% relative humidity and 16:8 h light to dark photoperiod), to which the potted plants were transferred two weeks prior to mite infestation.

### Spider mite DOG nomenclature

In the current study, we have given specific gene identifiers to the *DOG* genes of *T. urticae* and *P. citri.* The nomenclature of our *T. urticae DOGs* corresponds to the following gene identities in the ORCAE database (https://bioinformatics.psb.ugent.be/orcae/overview/Tetur) [30]; (***TuDOG1****-tetur01g00490*, ***TuDOG2****-tetur04g00150*, ***TuDOG3****-tetur04g08620*, ***TuDOG4****-tetur06g00450*, ***TuDOG5****-tetur06g00460*, ***TuDOG6****-tetur07g02040*,***TuDOG7****-tetur07g05930*, ***TuDOG8****-tetur07g05940*, ***TuDOG9****-tetur07g06560*, ***TuDOG10****-tetur12g04671*, ***TuDOG11****-tetur13g04550*, ***TuDOG12****-tetur19g03360*, ***TuDOG13****-tetur19g02300*, ***TuDOG14****-tetur20g01160*, ***TuDOG15****-tetur20g01790*, ***TuDOG16****-tetur28g01250* and ***TuDOG17****-tetur44g00140*). The nomenclature of the *P. citri DOGs* in this study corresponds to the following identities in the *Panonychus* transcriptomic data [31]; (***PcDOG5****- Pc_IDRCD16*, ***PcDOG6****- Pc_IDRCD9*).

### Confirmation of horizontal gene transfer and phylogenetic analysis of tetranychoid DOGs

Coverage plots for 17 *T. urticae DOG* genes were generated using the methodology described in Wybouw et al. [15] to confirm that they are real HGT genes and not genome contaminants. Three DOG genes (*TuDOG7*, *TuDOG10*, and *TuDOG11*) were also selected for PCR verification. A single PCR amplicon for each of these genes and their neighboring intron containing eukaryotic genes was generated using GoTaq G2 DNA polymerase (Promega). Primers sequences are listed in Table S1. The PCR conditions were initial denaturation at 95 °C for 2 min, 35 cycles of 95 °C for 30 s, 55 °C for 30 s,72 °C for 90 s and a final extension at 72 °C for 5 min. PCR products were purified using E.Z.N.A® Cycle pure kit (Omega Bio-tek) and sanger sequenced (LGC genomics, Germany). The sequenced data was analyzed in BioEdit version 7.2.

*T. urticae* DOG protein sequences were used as query in a tBLASTn search (E-value threshold of E-5) against transcriptomes of 69 of the 72 tetranychid species (excluding *T. urticae*, *Panonychus ulmi*, and *P. citri*) previously described in Matsuda et al. [32]. The *getorf* command from the EMBOSS package [33] was used to extract open reading frames (ORFs, translations between START and STOP, minimum length of 375 nucleotides) from the unique tBLASTn hits. Subsequently, ORFs were translated to protein sequences and *T. urticae* DOGs were used in a BLASTp search (*E*-value threshold of E-5) against these translated ORFs to identify tetranychoid DOGs. DOGs (> 125 AA) of *P. citri* and *P. ulmi* were previously identified [31] while those of *Brevipalpus yothersi* were identified by a BLASTp search (*E*-value threshold of E-5) against the manually curated proteome of *B. yothersi* [34] using *T. urticae* DOG proteins as queries. DOG proteins of each of the 73 tetranychoid species were filtered for identical sequences using the cd-hit program [35] with the “-c 0.98” option and DOGs of a given tetranychoid species as input. Filtered DOGs of each species were merged (496 sequences in total (see File S1), aligned using the online version of MAFFT version 7 with G-INS-I settings [36] and revealed that two DOGs (S_lesp_12131_2 and P_late_308_1) disturbed the alignment. These two DOGs were removed from the set of DOG sequences and a final alignment was generated in an identical way as the preliminary alignment. According to the online version of ModelFinder [37, 38] and using the Akaike Information Criterion, the WAG+G+F model was optimal for phylogenetic reconstruction. A maximum likelihood analysis was performed using RAxML v8 HPC2-XSEDE [38] on the CIPRES Science Gateway [39] with 1000 rapid bootstrapping replicates (“-p 12345 -m PROTGAMMAWAGF -f a -N 1000 -x 12345”). The tree was midpoint rooted and visualized using Iroki [40].

### *T. urticae* DOG transcript analysis in different life stages, resistant strains, and strains adapted/acclimatized to different host plants

Protein sequences of the 17 *T. urticae* DOGs [13] were aligned using the online version of MAFFT v7 [36] with default settings. The resulting alignment was used in a maximum likelihood analysis using RAxML v8 HPC2-XSEDE [38] on the CIPRES Science Gateway [39], with 1000 rapid bootstrapping replicates and protein model set to “auto” (“-p 12345 -m PROTGAMMAAUTO -f a -N 1000 -x 12345” option). The tree was midpoint rooted, visualized using MEGA 6.0 [41], and used to order *DOG* genes in the DOG expression heatmap. Microarray gene expression data of *T. urticae* populations resistant to acaricides and *T. urticae* lines adapted/acclimatized to different host plants were derived from previous studies [14, 22, 42–47] and gene expression analysis was performed as in Snoeck et al. [48]. In addition, we re-analyzed the microarray gene expression data of Bryon et al. [49] based on the methodology of Snoeck et al. [48] but using non-diapausing LS-VL as the reference. Fold changes for *T. urticae DOG* genes were extracted and a heatmap was constructed using the ComplexHeatmap version 2.4.3 package [50] in R. RNAseq reads of *T. urticae* stages (Illumina trimmed reads with a length of 60 bp) and *T. urticae* males/females (Illumina paired-end, strand specific 100 bp reads) were aligned as described in Wybouw et al. and Ngoc et al. [15, 51], respectively. RNAseq read counts per gene, based on the annotation of August 11, 2016, were obtained using the default settings of HTSeq 0.6.0 [52] with the “FEATURE” flag set to “exon” and the “ORDER” flag set to “pos.” For stage-specific RNAseq read count data, technical replicates data were collapsed using the DESEQ2 v. 1.28.1 package [53] in R, and genes with no read counts for any of the four stages were excluded from further analysis, while genes with no read counts for any of the four biological replicates were excluded from the male/female RNAseq read count data. Next, read counts were normalized by the method of trimmed mean of M-values (TMM) and log_2_ (counts per million (CPM)) were calculated using the edgeR version 3.30.2 package [54]. For the male/female RNAseq data, log_2_(CPM) values were averaged across biological replicates (four biological replicates/sex). Finally, log2(CPM) values were extracted for *T. urticae DOG* genes and a heatmap was constructed using the ComplexHeatmap version 2.4.3 package [50] in R.

### *T. urticae* DOG transcript analysis in response to tomato defense

To establish the transcriptional changes that JA-mediated defenses induce in mites, *T. urticae* adult females were transferred from common bean to 21-day-old tomato plants, CM and *def-1*. To obtain age-synchronized females, adult females were transferred from the stock cultures to detached bean leaves and removed after 48 h. Offspring was allowed to reach adulthood. About fifteen 2-day-old females were transferred to a tomato leaflet, and three leaflets per plant were infested (45 mites in total). Mites from 5 plants were pooled (max of 225 mites per pool) and constitute a biological replicate. Experiments were repeated 4 times in 4 consecutive weeks. Mites were frozen in liquid nitrogen and kept at − 80 °C until RNA extraction.

Total RNA from frozen mite samples was isolated using the RNeasy Mini Kit from Qiagen (Venlo, The Netherlands) following the manufacturer’s guidelines. One microgram of RNA was DNAse-treated, and 500 ng was used for cDNA synthesis. cDNA was diluted 10 times, and 1 μL of this dilution was used as template for a quantitative polymerase chain reaction (qPCR) using the Platinum SYBR Green qPCR-SuperMix-UDG kit (Invitrogen, Thermo Fisher Scientific, USA) and the ABI 7500 Real-Time PCR system (Applied Biosystems, Foster City, CA, USA). Primers used in qPCR experiments are provided in Table S1. The normalized expression (NE) data were calculated by the ΔCt method [55]. Using 18S (*tetur01g03850*) as the reference gene [56], NE of each target gene was compared using a nested ANOVA with “Mite Strain” and “Plant Genotype” were included as factors and “technical replicate” (i.e., two for each reaction) nested into the corresponding biological replicate (cDNA sample). Means of each group were compared by Fisher’s LSD post hoc test using PASW Statistics 17.0 (Chicago: SPSS Inc.). To plot the relative expression, NE-values and SEM were scaled to the treatment with the lowest average NE. To test statistical significance, an independent *t*-test was performed for each mite line, comparing DOG expression in defenseless tomato line (*def-1*) *vs* wild type (CM). *P*-values were corrected for false positives using Holm-Sidak method.

### *In situ* hybridization

In situ hybridization (ISH) was carried out to identify the *in vivo* localization of the transcription of *TuDOG1*, *TuDOG11*, and *TuDOG16* in *T. urticae*. We based our protocols on previous work [46]. RNA was extracted from a pool of 100-120 adult females of the London strain using the RNeasy plus mini kit (Qiagen)). Approximately 2 μg of RNA was used to synthesize cDNA with a Maxima First Strand cDNA Synthesis Kit (Thermo Fisher Scientific). Primers were designed using Primer3 [57] and amplified a fragment of approximately 300 bp (Table S1). After purification with an E.Z.N.A. Cycle Pure Kit (Omega Bio-tek, GA, USA), PCR products were cloned into pGEM-T plasmids (Promega) and transformed into *E. coli* DH5α cells using heat-shock method [58]. Based on colony PCR assays, eight positive colonies for *TuDOG1*, *TuDOG11*, and *TuDOG16* were grown in 5 mL LB broth (Duchefa Biochemie) at 37 °C with shaking at 250 rpm for 16 h. Plasmids from these liquid cultures were purified with an E.Z.N.A. Cycle Pure Kit (Omega Bio-tek, GA, USA). Insert orientations and nucleotide sequences were determined by Sanger sequencing (LGC Genomics, Germany). PCR assays were performed on the purified plasmids using pUC/M13 primers (Table S1) to generate amplicons that contain our approximately 300 bp insert flanked by the T7 and SP6 promoter sites. The cycling conditions were 95 °C for 2 min, 35 cycles of 30 s at 95 °C, 45 s at 57 °C, 1 min at 72 °C and 5 min at 72 °C. After verification by agarose gel electrophoresis, PCR products were purified using an E.Z.N.A. Cycle Pure Kit (Omega Bio-tek, GA, USA). An in vitro labeling reaction was performed using T7 or SP6 RNA polymerase (Roche), digoxigenin-uridine triphosphate (DIG-UTP, Roche), and the purified PCR product, to generate sense or anti-sense DIG-labeled probes. Probes were purified using Sigma Spin ^TM^ Sequencing Reaction Clean-Up Columns (Sigma) supplemented with hybridization buffer (50% formamide (Sigma), 2x SSC (Sigma), 1x Denhardt’s solution (Sigma), 200 μg mL^−1^ tRNA (wheat germ type V, Sigma), 200 μg mL^−1^ ssDNA (boiled salmon sperm DNA, Sigma), 50 μg mL^−1^ heparin (sodium salt, Sigma), 10% dextran sulfate (sodium salt, Sigma), and stored at −20 °C until use.

Adult female mites of the AT-London strain were collected in an Eppendorf tube with 30% sucrose in 1x PBS (0.85% NaCl, 1.4 mM KH_2_PO_4_, 8 mM Na_2_HPO_4_, pH 7.1) and kept at 4 °C for 1–2 h. Specimens were mounted in an Optimal Cutting Temperature (OCT) compound (Tissue-Tek; Sakura), sectioned into 7-μm thickness using a *Leica* cryostat CM1860, and then mounted on silanized slides. After air drying for 15 min, the slides were fixed in 4% formaldehyde at 4 °C for at least 30 min, then followed with washing in PBS (1 min), 0.6% HCl (10 min) and PBS with 1% TritonX-100 (2 min). After two times 30 s washes in PBS, slides were pre-hybridized in 100-150 μL of hybridization buffer at 52 °C for 30–60 min. Slides were covered with coverslips and hybridized overnight (20–24 h) at 52 °C in a closed container that contained 2x SSC. Coverslips were subsequently removed and the slides were washed in 0.2× SSC for 2× 30 min. Slides were subsequently rinsed shortly in TBS (100 mM Tris, pH 7.5, 150 mM NaCl) after which 1 mL of 0.1% BSA in TBS with 0.03% Triton X-100 was added on each slide followed by incubation at room temperature for 30 min. After the solution was discarded, the slides were incubated with the antibody mixture (anti-DIG-AP: BSA buffer = 1:500) at 37 °C for 1 h. Slides were subsequently washed three times in TBS, 0.05% Tween-20 for 5 min and washed in DAP-buffer (100 mM Tris, 100 mM NaCl, 10 mM MgCl_2_, pH 8.0), and in TBS, 0.05% Tween for 5 min. Fast Red/HNNP mix (Roche) was added to the slides, followed by incubation at room temperature for 30 min in the dark. After careful removal of the coverslip, slides were washed three times for 5 min in TBS, 0.05% Tween-20. The slides were mounted with antifade mounting medium (Vectashield) and covered with coverslips for further microscopic investigation (Nikon A1R fluorescence confocal microscope; emission at 500–530 nm and acquisition at 488 nm for spider mite auto-fluorescence and emission at 570–620 nm and acquisition at 561.7 nm for FastRed signal). Z-stacks were created using 4 slices with 2–3-μm distance between slices. All images were processed with Fiji and CorelDRAW Home & Student ×7.

### DOG silencing by dsRNA injection

To further investigate the functional importance of DOGs to host plant use, we selected two key *DOG* genes (*TuDOG11* and *TuDOG16*) for transcriptional silencing by dsRNA injection. Total RNA of 200 adult females of the MR-VP strain was extracted using RNeasy Plus Mini kit (Qiagen, Belgium), reverse transcribed using the Maxima First Strand cDNA synthesis kit (Thermo Fisher Scientific), and amplified using gene-specific primers (Table S1) that contain 23 bases of the T7 promoter. The PCR conditions were as follows; initial denaturation at 95 °C for 2 min, followed by 35 cycles of amplification (95 °C for 30 s, 60 °C for 30 s, 72 °C for 1 min) and a final extension at 72 °C for 5 min. PCR products were purified using E.Z.N.A. Cycle Pure kit (Omega Bio-Tek, USA), sub-cloned in pJET/Blunt 2.0 Vector, and transformed by heat shock into *E. coli* DH5ɑ component cells. Selection was performed on LB agar plates with 50 μg mL^−1^ carbenicillin. Positive colonies were confirmed by colony PCR and Sanger sequencing (LGC Genomics, Germany). To obtain pure and highly concentrated PCR template for dsRNA synthesis, a second PCR amplification step was performed on the plasmids. PCR products were purified using E.Z.N.A. Cycle Pure kit (Omega Bio-Tek, USA). The quantity and quality of the PCR products were assessed by DeNovix DS-11 spectrophotometer (DeNovix, USA) and by running an aliquot on a 2% agarose gel. Next, dsRNA was synthesized using Transcript Aid T7 High Yield Transcription kit (Thermo Fisher Scientific). After synthesis, dsRNA was purified by isopropanol precipitation [59]. The quantity and quality of dsRNA were assessed by a DeNovix DS-11 spectrophotometer (DeNovix, USA) and by running an aliquot on a 2% agarose gel. Injections of dsRNA were carried out as described by Dermauw et al. [60]. Briefly, 200 two-day old adult females of the MR-VP strain were immobilized on Alura red stained 2% agarose gel platforms for each treatment. Females were injected with 3 nL of a 1 μg μL^−1^ dsRNA solution targeting either *GFP*, *TuDOG11*, or *TuDOG16*. The dsRNA solution was injected into the ovary (near the third pair of legs) under a Leica S8 APO stereomicroscope using a Nanoject III injector (Drummond Scientific, USA). Needles used for injections were made from 3.5″ glass capillaries (Drummond Scientific, USA) using a PC-10 Dual-Stage Glass Micropipette Puller (Narishige, Japan) with 2-steps and following settings for steps 1 and 2 respectively: 101.1 and 96.4. Eight batches of 200 females were injected per dsRNA treatment. Each mite batch was transferred to a detached kidney bean leaf and allowed to recover. After 72 h, approximately 40–80 mites were collected per treatment and RNA was extracted using the RNeasy plus kit (Qiagen). RNA integrity was verified on 1% agarose gel after which cDNA was prepared from 1 μg RNA using the maxima cDNA synthesis kit (Thermo Fisher Scientific). qPCR assays were performed using GoTaq®qPCR master mix (Promega corporation). The thermal profile for qPCR was as follows: 40 cycles of 95 °C for 15 s, 55 °C for 30 s, and 60 °C for 30 s. Silencing efficiency was determined using qbase+ with ubiquitin (*tetur03g06910*) and glyceraldehyde-3-phosphate (*tetur25g00250*) as the reference genes (An M value of 0.6 was obtained, reflecting stability of the housekeeping genes). Primers for dsRNA synthesis and qPCR are outlined in Table S1. The remaining mites (about 60–100 mites per batch) were transferred to tomato leaves cv “Moneymaker.” Mortality and fecundity were scored at different time points (24, 48, and 72 h) on tomato. Mortality was defined as the percentage of dead mites at each time point relative to the total number of founding mites, whereas fecundity was defined as the total number of eggs divided by the surviving mites at each time point. To test statistical significance, a paired *t*-test was performed to compare each DOG treatment with GFP treatment using rstatix package in R (R development core team 2017) and GraphPad prism v.6.01 softwares.

### Functional expression of recombinant DOGs

The enzymatic abilities of spider mite DOGs were investigated by recombinant expression of some key DOGs in *E. coli*. To see whether the aromatic substrate ranges of *DOG* genes differed across species of the Tetranychidae family, we selected two DOGs (*PcDOG5* and *PcDOG6)* from the citrus red mite, *P. citri* that were specific to the *Panonychus* clade [31]. All expression constructs were designed using SnapGene® viewer v. 5.1.6 and added an N-terminal 6x His-tag for purification and detection. Signal peptides were predicted using SignalP v.5.0 [61] and removed from the final expression constructs to allow for cytoplasmic expression. The coding sequences for *TuDOG1*, *TuDOG11*, and *TuDOG16* were retrieved from ORCAE [30], codon optimized for expression in *E. coli* and produced by ATUM (Newark, CA) in the expression vector pJExpress 411. Additional DOGs constructs were designed from cDNA amplified from spider mite strains. The London strain was used to generate *TuDOG7* and *TuDOG15* constructs while the Lahijan strain was used to obtain *PcDOG5* and *PcDOG6* constructs. For cDNA preparation, total RNA was extracted from approximately 200 mites using RNeasy plus mini kit (Qiagen) and reverse transcribed using Maxima®first strand cDNA synthesis kit (Thermo Fisher Scientific). Full-length cDNAs (from start to stop codon) were amplified by a Phusion high fidelity DNA polymerase (Thermo Fisher Scientific). Primers sequences are listed in Table S1. The PCR conditions were 98 °C for 30 s, 30 cycles of 98 °C for 10 s, 60 °C for 30 s,72 °C for 30 s, and a final extension at 72 °C for 5 min. PCR products were purified using E.Z.N.A® Cycle pure kit (Omega Bio-tek), cloned into PJET 1.2/blunt vector, transformed into *E.coli* DH5α cells using the heat shock method and grown on LB agar plates substituted with 50 μg mL^−1^ carbenicillin. Five colonies per *DOG* gene were selected, grown in 5 mL LB broth with 50 μg mL^−1^ carbenicillin, plasmid extracted using the E.Z.N.A® plasmid mini kit II (Omega Bio-tek), and Sanger sequenced (LGC genomics, Germany). Based on the obtained sequences (File S2a), codon optimized constructs of *TuDOG7*, *TuDOG15*, *PcDOG5*, and *PcDOG6* without the signal peptide were generated by Genscript (The Netherlands), cut from the storage vector puC57 using NdeI and BamHI restriction enzymes, and cloned into the final expression vector PJExpress 411 (for codon optimized sequences, see File S2b).

Protein expression conditions were optimized in 5 mL liquid cultures to maximize protein yields. For large scale expression, *E. coli* BL-21 (DE3) cells were transformed by heat shock, grown in 1 liter LB broth with 50 μg mL^−1^ kanamycin at 37 °C and shaken at 250 rpm in a 311DS Environmental shaking incubator (Labnet international, USA) until OD_600nm_ = 0.8 was reached. After cooling the cultures to 16 °C, protein expression was induced with 4 mL of 100 mM isopropyl β-D-1 thiogalactopyranoside (IPTG) to a final concentration of 0.4 mM. To promote the incorporation of an iron cofactor into the recombinant DOG proteins, 3 mg of ferrous sulfate heptahydrate was added. The induced cultures were grown for an additional 24 h at 16 °C with shaking at 180 rpm in Innova 40/40R-benchtop orbital shaker (Eppendorf, Germany). Cells were harvested by centrifuging at 8000 g for 10 min. The pellet was stored at − 80 °C overnight, thawed and suspended in 100 mL lysis buffer (0.1 M PBS pH 7.5, 500 mM NaCl, 10 mM imidazole, 2% glycerol and 0.5 mM PMSF), and lysed by sonication on ice for 30 min. The lysate was centrifuged at 8000 *g* for 30 min and the supernatant passed through NiNTA column equilibrated with wash buffer (0.1 M PBS pH 7.5, 500 mM NaCl and 20 mM imidazole). Unbound proteins were washed off the column twice, first with the wash buffer containing 20 mM imidazole then with the wash buffer containing 30 mM imidazole. The protein was eluted with elution buffer (0.1 M PBS pH 7.5, 500 mM NaCl) containing increasing concentrations of imidazole between 50 mM and 200 mM. Following an SDS-PAGE, imidazole fractions containing the recombinant protein were pooled together, concentrated and desalted using Amicon®Ultra-15 centrifugal filter units (Sigma Aldrich). Protein concentration was determined using Pierce ^TM^ Coomassie (Bradford) protein assay kit (Thermo Fisher Scientific). The concentrated protein was stored at − 20 °C with addition of glycerol to a final concentration of 25%.

### Ferrozine assay

The ability of our recombinant DOGs to incorporate iron was verified using a modification of the ferrozine assay described by Ring et al. [62]. Briefly, 10 μM protein in 150 μL distilled water was hydrolyzed in an equal volume of acid digestion solution (a mixture of 1:1 ratios of 1.2 M HCl and 1.2 M ascorbic acid) at 60 °C for 3 h. The hydrolyzed protein was cooled to room temperature prior to adding 150 μL of iron chelating reagent (freshly prepared mixture of 5 M ammonium acetate, 2 M ascorbic acid, 6.5 mM ferrozine, and 13.1 mM Neocuproine). 210 µL of the reaction mixture was transferred into a 96 well plate, covered in foil and incubated at room temperature for 30 min. The absorbance at 562 nm was recorded and iron content calculated from iron standards of 0-20 μM prepared from Mohr’s salt subjected to the same hydrolysis and detection procedure as the samples.

### Spectrophotometry

As a first step to characterize the substrate range of our recombinant DOG enzymes, we defined their kinetic parameters using the model substrates catechol, 4-methyl catechol, and 4-chlorocatechol. All substrates were purchased from Sigma-Aldrich (Belgium). The reactions were carried out in triplicate in a 96 well UV-Star® Microplate (Greiner Bio-one), with a total reaction volume of 200 μL per well consisting of 0.1 M PBS pH 7.5, 1 μg, or 5 μg enzyme and varying substrate concentrations of 5–500 μM. The reaction was initiated by adding the aromatic substrate to a mixture of buffer and enzyme. The formation of muconic acids at 260 nm (catechol and 4-chlorocatechol) and 255 nm (4-methyl catechol) was followed for 5 min. For negative controls, the recombinant enzyme was omitted. The initial velocity (v_o_) with each substrate concentration was calculated using molar extinction coefficients of 16800 M^−1^ cm^−1^ for catechol, 14300 M^−1^ cm^−1^ for 4-methyl catechol [16], and 12400 M^−1^ cm^−1^ with 4-chlorocatechol [63]. Curves of initial velocity *vs* substrate concentration were plotted using Sigma Plot v.13 (Systat Software Inc., USA) and kinetic parameters were determined for each enzyme with the three model substrates. The total protein used in *k*_cat_ calculations was corrected for iron content using an iron factor calculated for each protein in the ferrozine assay (see above). As such, only the total amount of iron bound enzyme was used in the *k*_cat_ calculations.

### Oxygen consumption

We further investigated the substrate range of our recombinant DOGs using other more complex organic compounds in addition to the model substrates. All substrates were purchased from Sigma-Aldrich (Belgium) except trans-clovamide which was purchased from Cayman Chemical (The Netherlands). Enzymatic activity was tested against a total of 33 plant secondary metabolites, five model substrates, and two pesticides. All substrates are listed in Table S2. Using the Oxytherm+System (Hansatech, UK), ortho-cleavage (i.e., cleavage of the aromatic ring between two neighboring hydroxyl groups) was evaluated by recording oxygen consumption rates following incubation with the recombinant *T. urticae* DOGs. The electrode was prepared and calibrated to 25 °C before use. All reactions were carried out in three to four replicates in a total reaction volume of 1 mL, with the stirrer speed set to approximately 700 rpm (75% steps) for optimal oxygen circulation. Briefly, 984 μL of 0.1 M PBS pH 7.5 was added to the reaction chamber, stirred and the signal allowed to equilibrate for 90 s after which 5 μL of 20 mM substrate stock prepared in methanol was added and the chamber sealed from external oxygen. After signal equilibration for 90 s, 2 μL of boiled enzyme was injected into the chamber. Oxygen signal was recorded for 2 min at intervals of 10 s prior to injection of 9 μL active enzyme (3 or 5 μg). Oxygen consumption with the active enzyme was measured for 5 min. The oxygen consumption rate (OCR) was determined from the slope of the linear part of the graph after addition of active enzyme and corrected for background oxygen consumption with the OCR observed for the boiled enzyme control. Rates above 1 nmol mL^−1^ min^−1^ were scored as activity while lower rates were scored as background noise. A detailed graphical representation of the assay is provided in Figure S2. The OCRs for various substrates were compared and visualized using R package ggplot2 (R Development core Team 2017).

### Identification of reaction products by UPLC-MS

To confirm ortho-cleavage of the aromatic rings by *T. urticae* DOGs, a selection of eleven plant secondary metabolites that showed oxygen consumption using the Oxytherm+System (see above) was subjected to further metabolomic analysis by UPLC-MS to identify the reaction products (Figure S3). Analysis was carried out at the VIB Metabolomics Core Facility (Ghent, Belgium). Prior to metabolite analysis, all substrates were standardized to ensure their detectability using a similar protocol as described by Vanholme et al. [64]. Briefly, 20 μL of a 1-mM stock methanol solution was mixed with an equal volume of distilled water, and then 10 μL of the mixture was injected into the UPLC-MS system (Waters Corporation) and the detectability checked in both positive and negative modes. The metabolomics reactions were carried out in five replicates with either boiled or active DOG enzyme in a 1 mL reaction volume. Specifically, to a 2 mL sterile Eppendorf tube, 986 μL of 0.1 M PBS pH 7.5 was added, 9 μL active or boiled enzyme (a total of 3 μg) and 5 μL of 20 mM substrate stock. The tubes were loosely capped to allow oxygen diffusion and incubated at 25 °C with shaking at 300 rpm for 1 h. Reactions were stopped by adding 500 μL urea to a final concentration of 7 M. The samples were stored at − 20 °C until analysis. Reaction mixtures were analyzed on a UPLC-MS system as described in Vanholme et al. [64]. Briefly, 2 μL of the mixture was injected on a Waters Acquity UPLC® equipped with a UPLC BEH C18 column (130 Å, 1.7 μm, 2.1 mm × 50 mm) and coupled to a SynaptXS Q-Tof (Waters Corporation). A gradient of two buffers was used: buffer A (99/1/0.1 H_2_O/acetonitrile/formic acid, pH 3), buffer B (99/1/0.1 acetonitrile/H_2_O/formic acid, pH 3); 99% A for 1 min decreased to 50% A in 10 min (500 μL/min, column temperature 40 °C). The flow was initially diverted to waste for 0.5 min, and then to the MS equipped with an electrospray ionization source and lock spray interface for accurate mass measurements operating in negative ionization mode. The MS source parameters were capillary voltage, 2.5 kV; sampling cone, 40 V; source temperature, 120 °C; desolvation temperature, 550 °C; cone gas flow, 50 L h^−1^; and desolvation gas flow, 800 L h^−1^. The collision energy for the trap and transfer cells was 4 V and 2 V, respectively. For data acquisition, the dynamic range enhancement mode was activated. Full scan data were recorded in negative centroid sensitivity mode; the mass range was set between *m/z* 50 and 1200, with a scan speed of 0.1 s scan^−1^, with Masslynx software v4.1 (Waters). Leucin-enkephalin (250 pg μL^−1^ solubilized in water/acetonitrile 1:1 (vol:vol), with 0.1% formic acid) was used for the lock mass calibration with a scan time of 0.5 s. Data processing was done with Progenesis QI v2.4 (NonLinear Dynamics).

## Results

### The evolutionary history of spider mite DOG genes

First, the *T. urticae* genome assembly was studied in detail in order to verify that *DOG* genes indeed reside within the mite genome. Coverage plots for all the 17 *DOG* genes (File S3) showed equal coverage of DNA reads aligned to the assembled reference genome. In addition, single PCR amplicons of *TuDOG7*, *TuDOG10*, and *TuDOG11* with canonical intron containing eukaryotic genes were also generated (Figure S4 for the PCR amplified bands and Files S4, S5 and S6 for the DNA alignments of the three DOGs respectively). Together, these analyses provide sound evidence for the genomic integration of *DOG* genes in the *T. urticae* genome.

**Fig. 1:**
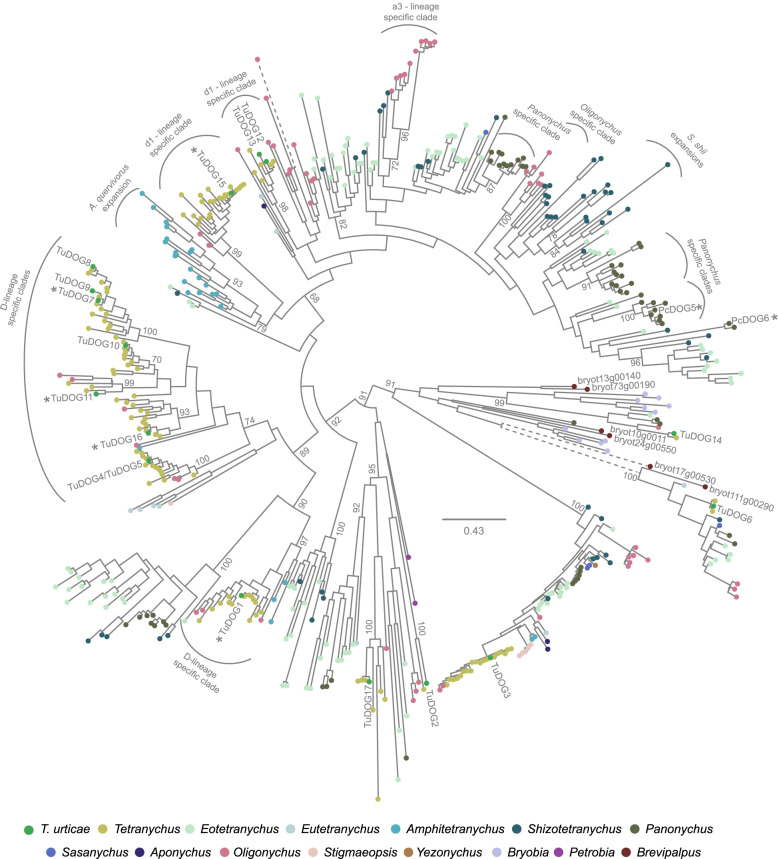
Phylogenetic reconstruction of horizontally acquired tetranychoid DOGs. Maximum-likelihood phylogenetic analysis of 494 tetranychoid DOGs. The 14 tetranychoid genera and *T. urticae* are color coded. Arches indicate lineage, genus, or species-specific clades. We named these lineage-specific clades (D, d1, or a3) in accordance with Matsuda et al. [32]. An asterisk indicates that the *T. urticae DOG* was functionally expressed in this study. Only bootstrap values above 65 and at phylogenetically relevant nodes are shown. The scale bar represents 0.43 substitutions per site. A dashed branch indicates that this branch was shortened to improve figure clarity. See File S1 for DOG sequences used for phylogenetic analysis and File S7 for an expanded version of the phylogenetic tree

Next, to obtain genome-wide insight in the evolutionary history of spider mite *DOG* genes, a phylogenetic analysis was carried out using transcriptomic and genomic data. *DOG* homologs were identified in all 72 spider mite species of the Tetranychidae family and in one false spider mite species of the Tenuipalpidae family (Fig. 1, File S7). Together with previous work that screened a wider range of metazoan genomes, these findings are in line with the previously proposed scenario that *DOG* genes restricted to bacteria and fungi were acquired by an ancestral mite species by horizontal gene transfer from fungi [13, 14, 16]. Current findings suggest that this transfer event occurred before the formation of the Tetranychidae and Tenuipalpidae families. Although caution is warranted for the comparison of gene numbers and orthologs detected in transcriptomic data [31, 32] and genomic data [13, 34], as recent gene duplications and genes with very low expression levels might be missed in transcriptomic data, some general patterns can be deducted from our analyses (Fig. 1, File S7). First, the number of identified *DOG*s does not appear to be correlated with the number of host plants the mite species is reported to feed on. For instance, the polyphagous *T. urticae* and *Oligonychus biharensis* have a similar number of *DOG*s as *Amphitetranychus quercivorus* and *Schizotetranychus shii*, which specialize on oak and *Castanopsis* sp., respectively (Table S3, spidermite web) [65]. Second, tetranychoid DOGs can either be lineage- (a3, d1, D) [32], genus- (e.g., *Panonychus* sp.*)* or species-specific (*A. quercivorus* and *S. shii*). Last, based on the phylogenetic analysis, we classified *T. urticae* DOGs into 13 orthologous clusters and for eleven clusters an orthologue could be detected in a specialist spider mite species (Fig. 1, File S1, Table S4). On the other hand, three of these eleven clusters contained duplicated *T. urticae* DOGs (*TuDOG12*/*TuDOG13*; *TuDOG7*/*TuDOG8*/*TuDOG9*; *TuDOG4*/*TuDOG5*) but whether these are duplicated in specialist spider mite species could not be clearly determined based on transcriptomic data alone.

### Transcriptional responses of T. urticae DOGs

**Fig. 2:**
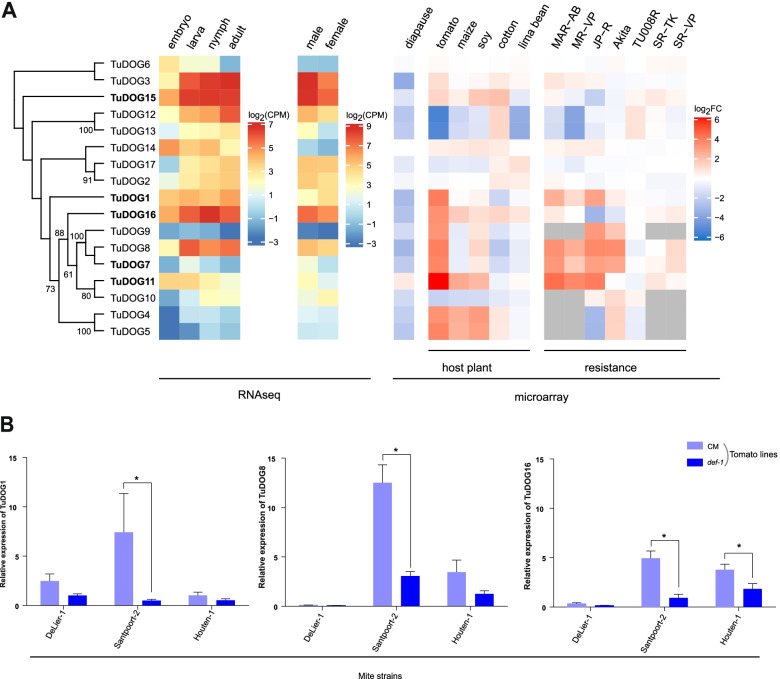
Host plant dependent *DOG* transcript accumulation in *T. urticae*. **A** Heatmap of *T. urticae DOG* transcript accumulation determined by RNAseq (left panel) or by microarray experiments (right-panel). For microarray experiments, expression data from either non-diapausing LS-VL adult females on common bean (diapause), London adult females on common bean (host plant data, MAR-AB, MR-VP, JP-R, Akita, and Tu008R), or LS-VL deutonymph females on bean (SR-VP, SR-TK) were taken as reference to calculate log_2_FC values. Gray boxes indicate that for a specific DOG gene no probes were included in the respective *T. urticae* microarray design, and hence expression could not be estimated. *T. urticae DOGs* in bold font were functionally characterized in this study. **B** Transcriptional response of *DOGs* to tomato defense. The expression of three *DOG* genes (*TuDOG1*, *TuDOG8*, and *TuDOG16*) was quantified by qPCR in three mite strains that differentially interact with jasmonic acid (JA) mediated tomato defense. Houten-1 induces the JA defenses of tomato and is resistant to it, Santpoort-2 induces JA-defenses and is susceptible to it, while DeLier-1 suppresses tomato defense. Error bars represent the standard error of the mean (*n* = 4). An asterisk indicates a significant difference (alpha = 0.05). Expression of the three *DOG* genes was significantly upregulated in the JA-defense susceptible strain Santpooort-2 feeding on the wild type plants compared to the *def-1* mutants (*P* values = 0.002, 0.0001, and 0.0001 with *TuDOG1*, *TuDOG8*, and *TuDOG16* respectively). The expression of *TuDOG16* was also significantly upregulated in the resistant strain Houten-1 feeding on the wild type plants compared to the *def-1* mutants (*P* value = 0.009). The three *DOGs* were not differentially expressed in the suppressor strain Delier-1 feeding on either wild type plants or *def-1* mutants (*P* values > 0.05)

Previously published life stage- and sex-specific RNAseq data were analyzed to investigate *DOG* expression in *T. urticae* (Fig. 2a). For thirteen of the 17 *T. urticae DOG* genes, we observed lower transcript levels in embryos than in any of the three feeding stages (larvae, nymphs, or adults), while for three *T. urticae* DOGs (*TuDOG6*, *TuDOG14*, and *TuDOG11*), levels were higher in the embryo than in any of the feeding stages. Noteworthy, the transcript levels of *TuDOG15*, *TuDOG3*, and *TuDOG16* were on average the highest of all the *DOG* genes during feeding stages (average log_2_CPM > 6, Table S5). *T. urticae DOG* genes showed similar transcript accumulation patterns in males and females, with *TuDOG15*, *TuDOG3*, and *TuDOG16* transcripts being the most abundant (log_2_CPM > 6) of all *DOG* genes in both males or females. All *T. urticae DOG* genes, except *TuDOG6*, *TuDOG14*, and *TuDOG11*, were significantly downregulated in adult diapausing females compared to non-diapausing females (Table S6) [49]. Multiple *T. urticae DOG* genes responded to the mites being transferred from common bean to another host plant (i.e., tomato, maize cotton, soy bean or lima bean), with six *DOG* genes being upregulated (log_2_FC > 1.5, adjusted *P*-value < 0.05) in mites after transfer to lima bean compared to 14 after transfer to tomato. Of note, *TuDOG16* was the only *DOG* gene that was always upregulated in spider mites upon transfer from common bean to another host plant, while *TuDOG11* displayed the highest level of upregulation (fold change) in mites transferred from common bean to tomato. *TuDOG11* was also the strongest upregulated gene in three pesticide resistant strains (MAR-AB, MR-VP and JP-R) (Fig. 2a, Table S6). Based on the phylogenetic tree (Fig. 1) and expression data (Fig. 2a), we selected some highly expressed lineage specific *DOG* genes for recombinant expression. The selected genes are marked with an asterisk in Fig. 1 and indicated in bold font in Fig. 2a. Three of the highly expressed lineage specific *DOG*s (*TuDOG1*, *TuDOG8*, and *TuDOG16*) were further investigated for their transcriptional response to tomato jasmonate defenses using three mite strains (Houten-1, Santpoort-2, and DeLier-1) that interact differently with jasmonic acid mediated tomato defenses [27, 28]. Houten-1 is a mite strain that resists the induced jasmonate defenses; Santpoort-2 is susceptible to these defenses; and DeLier-1 is susceptible to these yet is able to suppress them before taking effect [27, 28]. We found that in the resistant strain Houten-1, only *TuDOG16* expression was significantly different in wildtype CM tomato plants *vs def-1* mutants (*P* value = 0.009), suggesting that this DOG could have an important role in overcoming tomato jasmonate defenses. As previously noted, *TuDOG16* was also the only *DOG* differentially expressed in all mites transferred from bean to another host (Fig. 2a). Interestingly, all three *DOG*s were significantly upregulated in the JA-defense susceptible Santpoort-2 strain on CM tomato plants compared to *def-1* plants (*P* values = 0.002, 0.0001, and 0.0001 with *TuDOG1*, *TuDOG8*, and *TuDOG16* respectively). On the other hand, in the defense suppressor DeLier-1 strain none of the *DOGs* responded to the absence or presence of jasmonate defenses (*P* values > 0.05) (Fig. 2b, Table S7). Together, these data indicate that the expression of *TuDOG1*, *TuDOG8*, and *TuDOG16* is upregulated in spider mites that induced jasmonate defenses, especially in strains that are susceptible to these defenses.

### Spatial expression pattern of DOGs in T. urticae

**Fig. 3:**
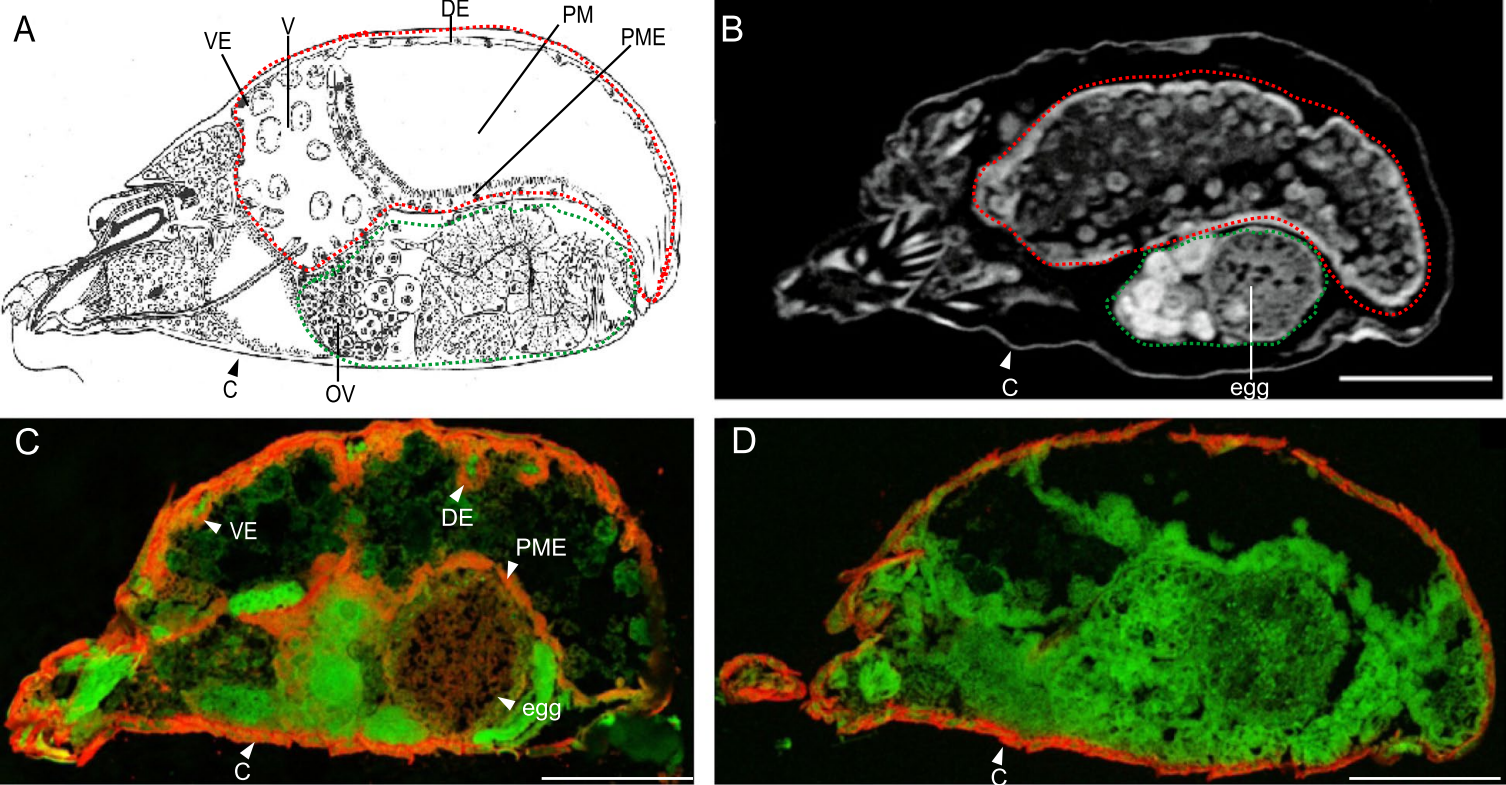
*In situ* localization of the transcription of *T. urticae DOG* genes. **A** A schematic representation of a mid-sagittal section of an adult *T. urticae* female redrafted from Alberti and Crooker [66] with the permission from Elsevier. The digestive and reproductive system is highlighted in dashed red and green lines, respectively. **B** A virtual mid-sagittal section obtained by a sub-micron CT scan of an adult *T. urticae* female. The internal morphology is as described by Jonckheere et al. [46]. **C** A DIG-labeled antisense probe for *TuDOG16* was used for hybridization, and the signal was developed using anti-DIG-AP and FastRed as substrate. The reaction product is visible as a red signal (indicating regions where DOGs are expressed) while the spider mite body shows green auto fluorescence. The cuticle stained red in both antisense and sense treatment, so we considered this as a false signal. **D** The control signal developed from a DIG-labeled sense probe for *TuDOG16*. C, cuticle; PME, posterior midgut epithelium; OV, ovary; PM, posterior midgut; V, ventriculus; VE, ventricular epithelium; DE, dorsal epithelium; scale bars: 100 μm

The localization of transcription of three *T. urticae DOG* genes (*TuDOG1*, *TuDOG11*, and *TuDOG16*) was evaluated using whole-mount *in situ* hybridization (ISH). In this ISH approach, the target transcripts are visible as a red signal, while the spider mite body shows green auto-fluorescence. Red auto-fluorescence of the cuticle was also seen in both antisense and sense (control) treatment (Fig. 3, Figure S5). Using mites that were adapted to tomato and overexpress *DOGs* [14, 23], ISH revealed that *TuDOG11* and *TuDOG16* were mainly transcribed in the digestive system, more precisely the posterior midgut epithelium, ventricular epithelium, and dorsal epithelium. Some staining was also seen in the developing eggs (Fig. 3). No clear staining was observed for *TuDOG1*, which could be as a result of poor probe design.

### Effect of DOG silencing on mite performance on a challenging host plant

**Fig. 4:**
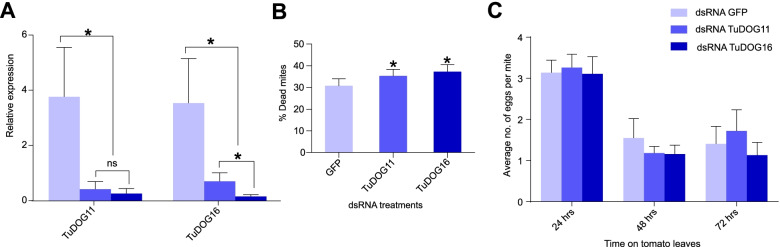
Silencing of *DOG* genes by RNA interference. **A** The relative expression of *TuDOG11* and *TuDOG16* was quantified by qPCR in adult female mites injected with dsRNA of either *DOG* or *GFP*. **B** Cumulative mortality. **C** Daily fecundity defined as the number of eggs laid by surviving mites after 24, 48, and 72 h on detached tomato leaves. Error bars represent the standard error of the mean (*n* = 8). Asterisks indicate a significant difference (alpha = 0.05). The relative expression of *TuDOG11* and *TuDOG16* after silencing was significantly different from mites treated with GFP dsRNA (*P* values = 0.005 and 0.006 with *TuDOG11* and *TuDOG16* respectively). Mortality was also significantly higher in *TuDOG* dsRNA-injected mites compared to GFP dsRNA-injected control mites (*P* values = 0.037 and 0.004 with *TuDOG11* and *TuDOG16* respectively). Daily fecundity was not affected by the dsRNA treatments (*P* values > 0.05)

To gain insights in DOG function and overall contribution to mite fitness, we used a reverse genetic RNAi approach to silence two tomato-induced *DOG* genes (see Fig. 2a), *TuDOG11* and *TuDOG16*, for which expression was shown in the digestive track of mites feeding on tomato. Micro-injection of 3 nL dsRNA that targets *TuDOG11* and *TuDOG16* resulted in a silencing efficiency of 89% (SE ± 9%) and 96% (SE ± 3%), respectively (Fig. 4a). However, we observed that each *TuDOG* dsRNA was not specific and silenced both *DOG* genes, limiting our ability to disentangle the effect of a single *DOG* gene. The relative expression of *TuDOG11* and *TuDOG16* after silencing was significantly different from mites treated with GFP dsRNA (*P* value = 0.005 and 0.006 with *TuDOG11* and *TuDOG16* respectively). Upon transfer to tomato, a significantly higher mortality was observed with *TuDOG* dsRNA-injected mites compared to GFP dsRNA-injected control mites (mortality of *TuDOG11 vs GFP*, *P* value = 0.037 and 0.004 with *TuDOG11* and *TuDOG16* respectively) (Fig. 4b, Table S8). *DOG* silencing did not significantly affect daily fecundity (*P* values > 0.05), although after 48 h on tomato *TuDOG* dsRNA-injected females laid a slightly lower number of eggs on average compared to GFP dsRNA-injected females (Fig. 4c). Of note, we observed high variations in the numbers of eggs deposited (SE-values ranged from 0.163 to 0.514) and hypothesize that this could have resulted from different degrees of wounding by needle injection.

### Functional characterization of spider mite DOG proteins

The coding sequences of five *T. urticae* and two *P. citri DOG*s were cloned into the expression plasmid pJExpress 411. As expression of the two *P. citri DOG*s was unsuccessful with the pJExpress 411 plasmid, the *P. citri DOG*s were recloned into pCold-SUMO expression plasmids (Takara Bio, France). Although this strategy improved the solubility, it failed to improve protein yield (≤ 1 mg). IPTG induction of the *T. urticae* constructs resulted in protein yields ranging between 2 and 12 mg from 1 L cultures (Table S9). All proteins were purified close to homogeneity as evidenced by a main band between 25 and 37 kDa on both SDS-PAGE and Western-blot with anti-His-tag primary antibody (Figure S6). Following chromatography purification, all proteins exhibited a dark red coloration on the NiNTA column. The red coloration remained visible in the final purified proteins that had a high yield (TuDOG11, TuDOG15, and TuDOG16) but was not apparent in the proteins of low yield batches (TuDOG1, TuDOG7, PcDOG5, and PcDOG6). Ferrozine assays confirmed that the purified DOG proteins contained iron, ranging from 0.3 to 1.0 nmol iron/nmol protein (Table S9), needed for catalytic activity.

The kinetic parameters of recombinant *T. urticae* DOGs were determined with spectrophotometric assays using model substrates catechol, 4-methyl catechol and 4-chlorocatechol. Due to the low protein yield and low activity observed with model substrates, the kinetic parameters of *P. citri* proteins were not characterized. TuDOG1 and TuDOG11 cleaved catechol with a higher efficiency as compared to 4-methyl catechol and 4-chlorocatechol (k_cat_/K_M_ 1.2-2.5 fold higher with catechol as the substrate for the two enzymes). TuDOG7, TuDOG15, and TuDOG16 showed preference for the substituted catecholates (Table 1). Under similar buffer and temperature conditions, four of the recombinant *T. urticae* DOGs had a much lower affinity to catechol (K_M_ 3-6 times higher) and 4-methyl catechol (K_M_ 2-5 times higher) compared to mTuIDRCD (TuDOG6) that was previously characterized by Schlachter et al. [16]. TuDOG1 had a relatively similar affinity to catechol as mTuIDRCD and cleaved this substrate with a 4 times higher efficiency as compared to that of mTuIDRCD under similar buffer and temperature conditions. TuDOG15 was the most efficient in cleaving 4-methyl catechol (k_cat_/K_M_ = 0.134 μM^−1^ min^−1^) and 4 chlorocatechol (k_cat_/K_M_ = 0.076 μM^−1^ min^−1^) compared to the other four recombinant *T. urticae* DOGs. Compared to bacterial homologs of *Rhodococcus opacus* and fungal homologs of *Candida albicans* and *Aspergillus niger*, the recombinant *T. urticae* DOGs were less efficient in cleaving these three model substrates (k_cat_/K_M_ values approximately 2 to 4 orders of magnitude lower) [9, 17, 18, 67]. In comparison to two hydroxyquinol dioxygenases (named NRRL3_05330 and NRRL3_02644) from *Aspergillus niger*, four of the recombinant *T. urticae* DOG enzymes were about 3–7 fold less efficient in cleaving 4-methyl catechol while TuDOG15 was about 1.4 fold more efficient than NRRL3_05330 in cleaving this substrate [67]. Additionally, TuDOG15 and NRRL3_05330 have relatively the same affinity towards 4-methyl catechol, 117 and 119 μM respectively.

**Table 1:** Steady state kinetic parameters (25 °C) of recombinant *T. urticae* DOG enzymes with catechol, 4-methyl catechol, and 4-chlorocatechol

DOG	Substrate	***K***_**M**_ (μM)	***V***_**max**_ (μM min^**−1**^)	***k***_**cat**_ (min^**−1**^)	***k***_**cat**_/K_**M**_ (μM^**−1**^ min^**−1**^)
**TuDOG1**	Catechol	15.2 ± 4.5	0.14 ± 0.01	2.269 ± 0.162	0.149 ± 0.036
	4-Methyl catechol	15.1 ± 2.7	0.12 ± 0.01	1.945 ± 0.162	0.129 ± 0.060
	4-Chlorocatechol	21.2 ± 7.4	0.08 ± 0.01	1.297 ± 0.162	0.061 ± 0.022
**TuDOG7**	Catechol	74.9 ± 30.9	0.42 ± 0.06	1.340 ± 0.191	0.018 ± 0.006
	4-Methyl catechol	62.2 ± 42.3	0.53 ± 0.23	1.691 ± 0.734	0.027 ± 0.017
	4-Chlorocatechol	115.2 ± 35.7	0.96 ± 0.23	3.062 ± 0.734	0.027 ± 0.021
**TuDOG11**	Catechol	64.9 ± 24.3	0.56 ± 0.04	3.202 ± 0.229	0.049 ± 0.009
	4-Methyl catechol	153.9 ± 24.6	0.58 ± 0.04	3.316 ± 0.229	0.022 ± 0.009
	4-Chlorocatechol	25.2 ± 4.6	0.09 ± 0.01	0.515 ± 0.057	0.020 ± 0.012
**TuDOG15**	Catechol	91.0 ± 21.2	0.09 ± 0.01	0.797 ± 0.089	0.009 ± 0.004
	4-Methyl catechol	117.4 ± 46.5	1.77 ± 0.37	15.678 ± 3.277	0.134 ± 0.070
	4-Chlorocatechol	72.0 ± 21.3	0.62 ± 0.09	5.492 ± 0.797	0.076 ± 0.037
**TuDOG16**	Catechol	51.0 ± 23.4	0.06 ± 0.01	0.483 ± 0.081	0.009 ± 0.003
	4-Methyl catechol	52.4 ± 19.6	0.14 ± 0.02	1.127 ± 0.161	0.022 ± 0.008
	4-Chlorocatechol	154.3 ± 20.9	0.31 ± 0.01	2.496 ± 0.081	0.016 ± 0.004

### Substrate diversity of T. urticae DOGs

**Fig. 5:**
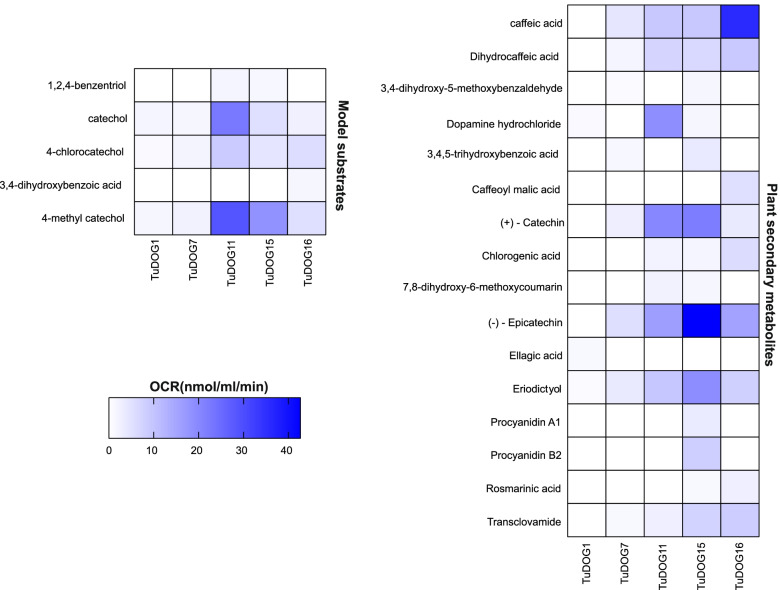
Substrate screening by oxygen consumption assay. Color map indicating oxygen consumption rates observed in a screening assay to identify substrates cleaved by spider mite DOGs. Five model substrates (left) and 16 of 33 plant secondary metabolites (right) screened were cleaved by our recombinant *T. urticae* DOGs. See TableS2 for a detailed list of the substrates screened. White boxes mean the substrate was not cleaved by that DOG while various shades of blue indicate ortho-cleavage of the substrate. The higher the color intensity, the more the OCR and therefore the higher the activity of that DOG on the said substrate. OCR; oxygen consumption rate

The ability of *T. urticae* and *P. citri* DOGs to cleave more complex substrates in addition to the classical model substrates was evaluated by measuring oxygen consumption using a Clark-type electrode. As DOG enzymes consume oxygen to cleave aromatic rings, oxygen depletion can be linked to the conversion of substrates to their dicarboxylic acid derivatives. First, we checked oxygen depletion with the three classical model substrates assayed earlier in spectrophotometry (catechol, 4-methyl catechol and 4-chlorocatechol) and two additional model substrates of microbial dioxygenases (hydroxyquinol and protocatechuic acid). In this setup, the three classical model substrates showed high OCR with the *T. urticae* proteins while the *P. citri* protein *pcDOG5* had a low OCR of 1.113 nmol mL^−1^ min^−1^ with 4-methyl catechol, confirming the finding from spectrophotometric assays. In addition, TuDOG11 and TuDOG15 showed activity towards hydroxyquinol while TuDOG16 was active on protocatechuic acid (Fig. 5).

Next, fourteen monocyclic and nineteen polycyclic compounds (Table S2) of plant origin containing adjacent hydroxyl groups were screened in oxygen consumption assays. Here, sixteen compounds resulted in oxygen consumption rates (OCRs) higher than 1 nmol mL^−1^ min^−1^ (Fig. 5), indicative of enzymatic activity towards a high number of newly uncovered DOG substrates. Interestingly, only six of the sixteen compounds cleaved by recombinant DOGs were monocyclic while the rest were polycyclic with varying degree of polymerization, ranging from dicyclic (e.g., transclovamide, chlorogenic acid, fraxetin and rosmarinic acid) to more complex structures (e.g., epicatechin, catechin, eriodictyol, ellagic acid and procyanidins A1 and B2). It is noteworthy that some of these polycyclic compounds namely eriodictyol, epicatechin, and catechin had similar or even higher OCRs compared to the model substrates, an indication that these complex plant metabolites could be better substrates for *T. urticae* DOGs. Our data revealed a low level of specificity of the *T. urticae* DOGs towards their substrates, as only 24% of all the metabolized substrates were cleaved by one particular DOG enzyme (Fig. 5). These include the model substrate protocatechuic acid cleaved by TuDOG16, ellagic acid (TuDOG1), caffeoyl malic acid (TuDOG16), and procyanidins A1 and B2 (TuDOG15). Procyanidin A1 was also cleaved by the *P. citri* protein pcDOG6, with an OCR of 1.691 nmol mL^−1^ min^−1^.

Last, to confirm that recombinant DOGs only cleave between adjacent hydroxyl groups, we also included two pesticides, all of which contain aromatic rings but lack adjacent hydroxyl groups. (i) 2,4-dichlorophenoxyacetic acid (2,4-D) is a phenoxy herbicide reported to be metabolized by dioxygensases [68, 69], and (ii) carbaryl is an insecticide also metabolized by dioxygenases [70]. None of them were cleaved by recombinant DOGs.

### Metabolite identification

**Fig. 6:**
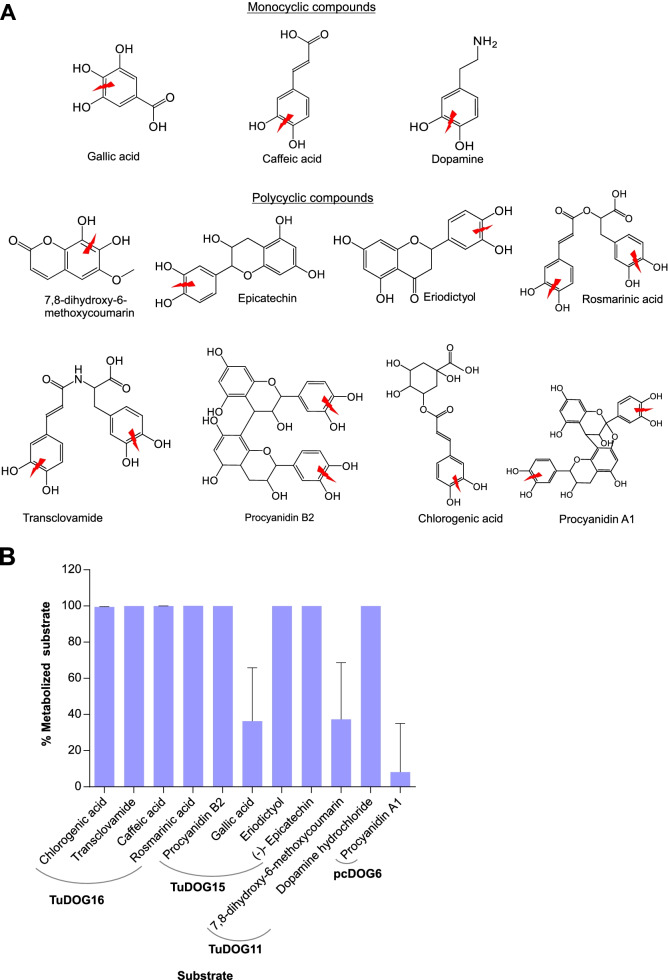
Confirmation of ortho-cleavage activity of *T. urticae* DOGs. **A** Chemical structures of the monocyclic and polycyclic catecholic compounds subjected to UPLC-MS. The ortho-cleavage positions are indicated with a red thunderbolt sign. **B** Percentage metabolized substrate as estimated from residual substrate in active and inactive enzyme treatments after 20 min incubation at 25 °C. The enzyme used with the various substrates is indicated under the curve. Error bars represent the standard error of the mean (*n* = 5)

As the quantification by OCR is an indirect method, the actual ortho-cleavage of the plant secondary metabolites was confirmed by UPLC-MS analysis. Eleven of the plant metabolites/DOG mixtures that showed oxygen consumption with the OCR measurements were subjected to UPLC-MS analysis. The substrate structures and predicted cleavage positions are shown in Fig. 6a. Since the substrate is continuously cleaved to a muconic acid derivative during the reaction with active enzyme, the percentage metabolized substrate was estimated from a comparison of the residual substrate in heat inactivated (control) and active enzyme treatments, revealing that eight of the eleven substrates were fully cleaved after 20 min of incubation with the active enzyme (Fig. 6b, Table S10). To confirm cleavage, metabolites in the form of muconic acid derivatives were detected using mass spectrometry. Muconic acids are important intermediates towards the formation of succinyl-CoA and acetyl-CoA, the final products of the β-KAP that converts toxic aromatic compounds into safe metabolites of the tricarboxylic acid cycle [6]. Additionally, their structures can be predicted based on the cleavage position in the catecholic ring of the aromatic compound, allowing for their detection in mass spectrometry. All ortho-cleavage reactions occurred at the predicted positions, with the predicted muconic acid derivatives being formed as confirmed by structure elucidation with the UPLC-MS. Interestingly, recombinant DOGs were able to cleave transclovamide and procyanidins at the two possible positions containing adjacent OH groups, thus yielding multiple metabolites. TuDOG16 assays yielded two metabolites (detected at 390.08 and 422.07 *m/z*) with transclovamide as substrate and TuDOG15 assays also yielded two metabolites (detected at 609.12 and 641.11 *m/z*) for procyanidins B2. Although at low OCR, pcDOG6 cleaved procyanidin A1 at the two possible positions simultaneously, yielding three different metabolites (two isomers detected at 607.11 *m/z* and a third metabolite detected at 639.10 *m/z*). All UPLC-MS peaks and chemical structures of the detected products are provided as a supplemental figure (Figure S3).

## Discussion

The complexity of plant-herbivore interactions is considered to be shaped by coevolutionary arms-races between plants and herbivores [71, 72]. Plants have evolved strategies to defend themselves against pests, including signaling pathways that can activate defenses specifically upon attack and establish a plethora of physiological responses that decrease their palatability. These include the accumulation of defense metabolites such as inhibitors of herbivore digestion and a wide range of toxins and antifeedants [73, 74]. Herbivore feeding damage also induces structural changes that serve to repair wounds or to reinforce tissues and often linked to lignin biosynthesis which takes place downstream of the phenylpropanoid pathway. Many early lignin precursors (e.g., caffeic, ferulic, and chlorogenic acids) can also function as defense compounds or have a role in defense signaling [19]. The ability of the feeding herbivore to cope with these compounds is therefore paramount for its survival and reproductive success. Overall, arthropods have developed a myriad of defense strategies, ranging from insensitivity of target-proteins and receptor sites (pharmacodynamic mechanisms), to detoxification, sequestration, and secretion (pharmacokinetic mechanisms) [75–77]. A number of enzyme families have been well-studied for their role in detoxification in arthropods, including those in spider mites. Previous studies have suggested that the evolution of a herbivorous generalist lifestyle might be linked to the expansion of several detoxification enzyme families, including cytochrome P450 mono-oxygenases, carboxylcholinesterases, glutathione-S-transferases, UDP-glycosyltransferases, and xenobiotic transporters [13, 14, 23, 78]. Surprisingly, a number of horizontally transferred genes were also uncovered in the *T. urticae* genome and are suggested to play a crucial role in the spider mite’s adaptation potential [13–15]. This includes *DOG* genes that encode enzymes which were suggested to directly ortho-cleave aromatic defense compounds of the host plant [14, 16], a metabolic activity unique in animals.

In this study, we used multiple complementary approaches to gather evidence for the involvement of DOGs in the xenobiotic metabolism of spider mites. First, a phylogenetic analysis provided evidence that the horizontal gene transfer event occurred before the split of Tetranychidae and Tenuipalpidae families (Fig. 1). We identified two clades containing DOGs from both mite families that could be more ancestral and represent a good candidate set to study the initial enzymatic abilities of the transferred *DOG* gene. Lineage specific expansion was also observed with *DOG* genes, most notably in the genus *Tetranychus*, where these patterns are also seen in other detoxification gene families [76, 79]. It is remarkable that an ortholog of *TuDOG3* could be found in nearly all (66/70) Tetranychinae species, suggesting this gene is highly conserved and could play an essential role in the (eco)physiology of this genus. However, the identification of this gene in the transcriptomes of so many species could also be related to a relatively high transcript abundance, which is at least the case for *T. urticae* (see Fig. 2a). The gene expression data in Fig. 2a further revealed that only three *DOG* genes (*TuDOG6*, *TuDOG11*, and *TuDOG14*) are highly expressed exclusively in the embryos, suggesting a conserved role unrelated to feeding/diet for these *T. urticae DOG* genes especially for *TuDOG6* and *TuDOG14* which were not lineage specific. Previous studies have suggested the involvement of extradiol ring cleavage dioxygenases in the development process by cleaving aromatic amino acids [80]. As such, the observed increase in expression level of the three dioxygenases could be an indication of a role in embryonic development, although this would be extraordinary for an intradiol enzyme. Furthermore, *in situ* hybridization also localized *TuDOG11* and *TuDOG16* to the egg before cell division, potentially indicating maternal import of DOG mRNA in the developing oocyte. This could either be consistent with a role in early development but also could serve as maternally provided protection strategy. This was recently also suggested for cytochrome P450 mono-oxygenases (CYPs), well-known detoxification enzymes, where maternally inherited CYP activity in the eggs was correlated with acaricide resistance in the egg [81]. The three dioxygenases were also highly expressed in diapausing mites that are characterized by an increased carotenoid biosynthesis [49, 82]. As previous studies have reported the involvement of extradiol dioxygenases in the metabolism of carotenoids in plants, fungi, and bacteria [83–85], the observed increase in *DOG* expression could point to a role in carotenoid metabolism in the diapausing mites. The remaining *DOG* genes were more highly expressed in the feeding stages (larvae, nymph, and adult) of mites feeding on different host plants than in the non-feeding stages, which makes a role in digestion and/or detoxification processes more likely. In this study, transcriptional responses of different spider mite strains feeding on wild-type tomato and a tomato mutant impaired in JA-induced defenses indicate that upregulation of some *DOG*s depends on JA-responsive compounds in the host plant and suggests that spider mites may do so to directly detoxify compounds after ingestion. In support of this idea, the *in situ* hybridization localized *DOG*s mainly in the gut (Fig. 3), also suggesting a primary function in detoxification.

We observed that all five recombinant DOGs exhibited activity against at least three of the five tested classic model substrates (catechol, 4-methyl catechol, 4-chlorocatechol, hydroxyquinol and protocatechuate). Schlachter et al. [16] observed that *TuDOG6* cleaves catechol and 4-methy catechol with much less efficiency than the bacterial and fungal homologs. A similar low efficiency was observed with the five *T. urticae* DOGs in our enzymatic assays, suggesting that catechol and 4-methyl catechol might not be the primary substrates of *T. urticae* DOGs. We therefore performed a high throughput substrate screening with an oxygen consumption assay to identify other substrates of *T. urticae* DOGs. We screened a panel of 33 plant metabolites as potential substrates, selected based on their presence in *T. urticae* infested plants, their reported toxicity in arthropods [86–90] and/or since they are plant metabolites that possess two adjacent hydroxylated carbons (ortho) in the chemical structure. Of the 33 tested metabolites, 16 gave rise to detectable and reproducible oxygen consumption rates (OCR), ranging between 1 to 40 nmol mL^−1^ min^−1^ (Fig. 5). Ortho-cleavage in 11 of the 16 detectable substrates was confirmed with UPLC-MS, providing first evidence that this family of DOGs can metabolize a plethora of natural substrates besides the few model substrates. Although formal evidence of a shift in toxicity between compounds and their ring-cleaved metabolites should be provided, this was technically not feasible. Nevertheless, it is clear that splitting an aromatic ring has a huge effect on the stability and the potential interactions of these molecules with their molecular targets. The wide array of substrates observed with the five recombinant *T. urticae* DOGs is surprising, and these results are in sharp contrast to the restricted enzymatic capabilities of DOGs of microbial organisms. We therefore hypothesize that *T. urticae* DOGs, after the transfer event from a fungal donor species, have evolved to metabolize a wide range of structurally unrelated plant metabolites. Indeed, we observed activity on compounds ranging from simple monocyclic organic compounds such as hydroxyquinol, caffeic and gallic acids, to complex polycyclic organic compounds such as phenolics (chlorogenic acid and rosmarinic acid) and flavonoids (epicatechin, eriodictyol, catechin and procyanidins), with an even higher efficiency than the classic model substrates catechol and substituted catechols. These results are consistent with the TuDOG6 (mTUIDRCD) structure as previously characterized by Schlachter et al. [16]. Their study revealed a relatively large opening at the active site that might accommodate complex substrates as compared to the bacterial DOGs that usually have a smaller active site. Enzyme activity was also observed with dopamine, a plant growth regulator that responds to biological stressors [91]. TuDOG16 showed high enzymatic activity for cleaving caffeic acid and chlorogenic acid (Figs. 5 and 6), both of which are well-known tomato defense compounds against a wide range of insect herbivores and whose accumulation in tomato leaves is induced by spider mite feeding [92, 93]. Even though most substrates were cleaved by more than one of the recombinant *T. urticae* DOGs, substrates like procyanidins (A1 and B2) and caffeoyl malic acid were only cleaved by *TuDOG15* and *TuDOG16* respectively. Previously, only fungal DOGs of plant pathogenic/associated fungi were reported to have the ability to degrade plant procyanidins [24, 94]. Caffeoyl malic acid on the other hand is degraded by *Streptomyces sp.*, a bacterial species capable of degrading a variety of phenylpropanoids [19]. The observation that horizontally acquired *T. urticae* DOGs of likely fungal origin degrade natural plant compounds strengthens the hypothesis that horizontal gene transfer is a driving force in the evolution of arthropod herbivory [95]. Within the context of benefiting from the enzymatic abilities of microbial organisms, it is interesting to note that a fungal DOG was found to play a central role in the association between the bark beetle *Ips typographus* and the fungus *Endoconidiophora polonica*. The beetle is a vector of the fungus and both attack Norway spruce. When feeding, the beetle induces an accumulation of defensive organic compounds that are subsequently cleaved by intradiol catechol dioxygenases of the fungus that utilizes them as a carbon source, thus enabling the bark beetle to escape the defense compounds of Norway spruce [94]. Transcriptional silencing of *T. urticae DOGs* further supports a role in host plant utilization. RNAi-mediated silencing of *TuDOG16* via dsRNA injection significantly affected the survival rate of *T. urticae* when transferred to tomato (Fig. 4). Together, these findings suggest that *TuDOG16* is very important for the mite’s ability to cope with the tomato defensive chemistry, possibly detoxifying caffeic and chlorogenic acids produced by the plant in response to spider mite attack. On the other hand, the omnipresence of DOGs in tetranychid species might also point towards a function for these enzymes across diverse plant hosts. All tetranychid mite species damage plant cells by piercing the cell wall with its stylets [96] and plant cells are known to initiate lignin production upon cell wall damage [97]. An increase in lignin production might in turn result in an increase in phenylpropanoid pathway intermediates, including 5-hydroxy-ferulic-acid and caffeic acid [98, 99]. Such catecholic compounds were often shown to exhibit defensive properties [100]. They are produced throughout the phenylpropanoid pathway and are believed to be converted into quinones by plant polyphenol oxidases (PPOs) in the herbivore gut. This is a two-component defense system where the plant accumulates herbivore-induced PPOs in vacuoles and *ortho*-dihydroxyphenolics in the cytosol, keeping them compartmentalized until they are ingested by a feeding herbivore and mixed in the gut. There is evidence that quinones generated by the PPOs after mixing them with these compounds damage the herbivore gut [101]. Interestingly, PPOs are among the best documented mite- and jasmonate-inducible enzymes in plants such as tomato [102]. Their expression is much lower in *def-1* [103] and they act on catecholic compounds [101] as do the DOGs. Therefore, we hypothesize that some DOGs may compete with plant PPOs in the mite gut to divert the production of dangerous quinones by PPOs towards the relatively harmless aliphatic acids. The accumulation of *ortho*-dihydroxyphenolics such as 5-hydroxy-ferulic-acid and caffeic acid has been previously observed in mite infested leaves [92] and tetranychid DOGs might then be key in neutralizing these compounds directly, or indirectly by substrate competition with PPOs in the herbivore gut [104].

Although some *DOG*s were highly expressed in pesticide resistant strains (Fig. 2a), the two pesticides tested in this study (2,4-D and carbaryl) were not metabolized, reinforcing the notion that DOGs act on aromatic rings with two adjacent hydroxyl groups. Given that in polyphagous herbivores and spider mites such as *T. urticae* in particular, large coordinated responses in gene expression were observed in response to pesticide selection and host plant use [14], we hypothesize that the observed increased expression of *DOG*s is probably a result of co-regulation in a more general stress response. Described coordinated responses have included genes involved in xeno-sensing, detoxification, and transport [14, 23] and is likely that DOGs further metabolize pesticides after the introduction of additional hydroxyl groups by CYPs. The DOG-mediated catabolism of catecholic intermediates formed downstream of the activation steps is typical for bacterial β-ketoadipate pathway, for example, the degradation of the herbicide 2,4-D that is first converted to 3,5-dichlorocatechol [68] or hydroxyquinol [69]. The insecticide carbaryl is first converted to 1,2-dihydroxynapthalene [70] prior to ortho-cleavage by bacterial DOGs.

To see whether substrates of DOG proteins differ between species of the Tetranychidae, we also expressed two DOGs (PcDOG5 and PcDOG6) from the citrus red mite (*Panonychus citri*), a citrus specialist, that were specific to the *Panonychus* clade (Fig. 1) [31]. Unfortunately, we experienced difficulties in obtaining high yield pure recombinant protein for these genes. Nevertheless, oxygen consumption assays showed activity on procyanidin A1 with PcDOG6. UPLC-MS analyses with PcDOG6 and procyanidin A1 also identified three metabolites formed from ortho-cleavage of the two hydroxyl positions present in procyanidin A1. Procyanidins function in many plants, including citrus, in defense pathways against biotic stress [105, 106]. Given that they are synthesized from polymerization of catechin or epicatechin units, we also expected activity on these compounds as the *T. urticae* DOGs were active on these subunits as well as on procyanidins but this was not the case, possibly due to low activity of the two *P. citri* proteins. Nevertheless, as both *TuDOG15* and *PcDOG6* can metabolize procyanidin A1, we can conclude that DOGs from different tetranychid species can act on the same substrates, while establishing their degree of specificity should be a subject of future study.

Variations in the observed enzymatic properties (iron content and low activity with some proteins) could be attributed to the bacterial expression system we used for their production. Spider mites and fungi are eukaryotic and hence recombinant protein production in a prokaryote, albeit codon optimized, may suffer from misfolding or other post translational differences. Producing mite DOGs in a eukaryotic expression system, for example using insect cell lines, may result in enzymes that exhibit a less variable activity and that provide a more apparent substrate-specific contrast. Additionally, in bacteria and fungi, DOGs often consist of heteropolymers [17, 67, 107–109]. We did not investigate this property for the recombinant DOG enzymes, as the first structure elucidation of TuDOG6 (named mTUIDRCD) by Schlachter et al. [16] revealed that this particular *T. urticae* DOG is active as a monomer. Co-crystallization of spider mite DOG enzymes with their substrates would be a step forward in understanding the substrate-enzyme interaction dynamics of these DOGs.

## Conclusion

In this study, we examined the evolutionary history of *DOGs* and deduced that *DOG* genes were horizontally acquired before the formation of the (phytophagous) Tetranychidae family. Based on phylogeny and expression profile, a selection of tetranychid *DOGs* was expressed in *E. coli*, purified and functionally characterized with oxygen consumption assays while ring cleavage was confirmed by UPLC-MS. We show here that spider mite DOGs metabolize a wide variety of complex organic phytochemicals, a unique property for this class of enzymes. Substrates include many plant metabolites associated with inducible defenses, suggesting that DOGs function as detoxification enzymes. This hypothesis was re-enforced by the gut specific expression of some DOGs, their JA-dependent expression, and their effect on spider mite survival on tomato when silenced by RNAi.

## Supplementary Information

**Additional file 1: File S1.** DOG sequences used for phylogenetic analysis. Format: FASTA, examination of this file requires the Bioedit program available at http://www.mbio.ncsu.edu/BioEdit/bioedit.html.

**Additional file 2: File S2a.** Alleles of *TuDOG7*, *TuDOG15* as picked from London strain of *T. urticae* cDNA and those of *PcDOG5* and *PcDOG6* as picked from Lahijan strain of *P. citri* cDNA. Format: FASTA, examination of this file requires the Bioedit program available at http://www.mbio.ncsu.edu/BioEdit/bioedit.html.

**Additional file 3: File S2b.** Codon optimized sequences of *TuDOG7*, *TuDOG15*, *PcDOG5* and *PcDOG6* as ordered from Genscript (The Netherlands) without the signal peptide sequence. Format: FASTA, examination of this file requires the Bioedit program available at http://www.mbio.ncsu.edu/BioEdit/bioedit.html.

**Additional file 4: File S3.** Coverage plots of 17 *T. urticae* DOG genes and their surrounding regions in the genome of *Tetranychus urticae.* Gene models of DOG genes and their neighboring genes are depicted as follows: large and small orange boxes represent coding sequences and untranslated regions, respectively, whereas introns are shown as connecting lines between the boxes. (+) and (-) represent the forward and reverse strand, respectively. Underneath the gene models of *TuDOG7, TuDOG10* and *TuDOG11*, the length and position of an amplicon obtained by PCR (Fig. S4) is indicated with a red line. Next, coverage plots of Illumina-reads (‘“Illu.’”) from adult *T. urticae* polyA selected RNA [13] and from genomic DNA sequencing of the EtoxR (‘“Etox’”) and London(‘“Lon’”) strain of *T. urticae* [13, 110] are shown below the gene models. The Illumina reads coverage plots are followed by a coverage plot of Sanger (‘“San.’”) reads from genomic DNA sequencing of the London strain [13] and by an alignment of these Sanger reads with the *T. urticae* genome of the London strain. Paired-end Sanger reads for which both reads are mapped in or extend nearby the indicated region are denoted by thin lines to show pair connections [13] (see [43], for mapping details).

**Additional file 5: File S4.** DNA alignment of *TuDOG7* with a neighboring eukaryotic gene (*tetur07g05920*) identified as guanylate kinase. Format: FASTA, examination of this file requires the Bioedit program available at http://www.mbio.ncsu.edu/BioEdit/bioedit.html.

**Additional file 6: File S5.** DNA alignment of *TuDOG10* with a neighboring eukaryotic gene (*tetur12g01070*) identified as glutaminyl peptide cyclotransferase. Format: FASTA, examination of this file requires the Bioedit program available at http://www.mbio.ncsu.edu/BioEdit/bioedit.html.

**Additional file 7: File S6.** DNA alignment of *TuDOG11* with a neighboring eukaryotic gene (*tetur13g03880*) identified as SSUH2 homolog isoform X2. Format: FASTA, examination of this file requires the Bioedit program available at http://www.mbio.ncsu.edu/BioEdit/bioedit.html.

**Additional file 8: File S7.** Detailed phylogenetic analysis of tetranychoid DOGs (rectangular representation). For abbreviation of species names and sequences see Table S3. Format: SVG, examination of this file requires a web browser e.g. Mozilla Firefox or Google Chrome.

**Additional file 9: Table S1.** Primers. Primers used in qPCR to quantify the transcriptional response of DOGs to tomato defense (sheet1-host transfer primers), Primers used to synthesize *in situ* probes (sheet 2-in situ primers), primers used in RNAi for dsRNA synthesis and qPCR (sheet 3-RNAi primers), primers used to amplify *TuDOG7*, *TuDOG15*, *pcDOG5* and *pcDOG6* gene alleles from a cDNA pool (sheet 4-DOG allele selection primers) and primers used for HGT verification of *TuDOG7, TuDOG10 and TuDOG11*(sheet 5-DOG-HGT verification primers).

**Additional file 10: Table S2.** Compounds used in the substrate screening assay by oxygen consumption using a Clark-type electrode. All substrates were purchased from Sigma Aldrich (Belgium) except Transclovamide (Cayman Chemical, Netherlands). 20 mM stocks were freshly prepared in either methanol or DMSO prior to the assays.

**Additional file 11: Table S3.** Number of *DOG* genes identified in tetranychoid species and the number of hosts these tetranychoid species have been reported on. **Table S4.** Orthologous tetranychoid DOG clusters containing *T. urticae DOG* genes. **Table S5.** log2CPM values of *T. urticae DOG* genes across different stages [13] and in *T. urticae* males or females [51]. **Table S6.** log2FC of *T. urticae DOG* genes in diapausing females, host plant adapted/acclimatized lines and in acaricide resistant strains.

**Additional file 12: Table S7.** Calibrated normalized relative quantity (CNRQ) data used to determine the relative expression of *TuDOG1, TuDOG8* and *TuDOG16* genes in spider mites strains interacting differently with the Jasmonic acid defense.

**Additional file 13: Table S8.** Calibrated normalized relative quantity (CNRQ) data used to determine the silencing efficiency of *TuDOG11* and *TuDOG16* genes following injection with dsRNA. (sheet 1). Fitness (Fecundity, mortality and survival) data collected from RNAi injections experiments (sheet 2).

**Additional file 14: Table S9.** Enzyme characteristics. Protein yields were estimated from protein concentrations determined using Bradford assay. Iron content was estimated using ferrozine assay with a self-developed Fe^3+^ standard curve. All purified DOG proteins had good enough yields, with the lowest yields in PcDOG5 and PcDOG6. All proteins were able to bind varying amounts of iron. The errors in iron content represent standard deviation of the mean (n=4).

**Additional file 15: Table S10.** UPLC-MS data generated for recombinant DOG enzymes. The retention time, mass to charge ratios (m/z) and peak areas of the substrates and reaction products formed after *in vitro* incubations with recombinant DOG enzymes are shown. The reactions were carried out in 5 replicates, with boiled enzyme controls indicated as C1-C5 and the active enzyme reactions indicated as T1-T5 alongside the tested substrate.

**Additional file 16: Figure S1.** Chemical structures of the three products formed from funneling reactions utilized by microorganisms in the breakdown of organic compounds. Catechol and protocatechuic acid degradation proceeds via the β-ketoadipate pathway while hydroxyquinol is formed from protocatechuate and degraded in an alternative pathway found in some bacteria and fungi.

**Additional file 17: Figure S2.** Schematic of oxygen consumption assay with Clark-type electrode. (A) The Clark type electrode reaction chamber. The rubber seal replaces the microsyringe after injection of the various components into the system. It serves to seal the chamber from external oxygen. (B) A zoom into the electrode unit. The platinum cathode is surrounded by a well that serves as a reservoir for electrolyte solution (50% potassium chloride). During the reaction, the electrolyte is ionized and initiates a current flow from anode to cathode. The current is equivalent to the oxygen concentration in the media. (C) Typical oxygen consumption curve observed when there is substrate cleavage. (D) The curve observed in the absence of substrate cleavage. In the absence of substrate cleavage, catechol was added at the end of the reaction as a control for activity.

**Additional file 18: Figure S3.** Spectra of selected substrates and their metabolites as detected in negative mode by UPLC-MS. Black peaks represent the substrate while red peaks depict the metabolites. Structures of both the substrate and the metabolite are as shown with their mass to charge ratios. The DOG used in the assay is indicated in blue. A thunderbolt sign shows the ortho-cleavage position.

**Additional file 19: Figure S4.** PCR amplicons of *TuDOG7, TuDOG10* and *TuDOG11* and their neighboring intron containing eukaryotic genes.

**Additional file 20: Figure S5.***in situ* localization of *T. urticae DOG* genes (*TuDOG11* and *TuDOG3*). A DIG-labelled antisense probe was used for hybridization and the signal was developed using anti-DIG-AP and FastRed as substrate. The reaction product is visible as a red signal (the cuticle got red staining in both antisense and sense treatment so we consider that as false signals) while the spider mite body shows green auto fluorescence. Abbreviations: C, cuticle; PME, posterior midgut epithelium; PM, posterior midgut; V, ventriculus; VE, ventricular epithelium; Scale bars: 100 μm.

**Additional file 21: Figure S6.** Stain-free SDS-PAGE (panel a) and western blot (panel b) of purified recombinant *T. urticae* DOGs. The 6x His tagged proteins are between 25-37 kDa.

## Data Availability

All datasets supporting the conclusions of this article are included within the article and its additional files. Additionally, TuDOG protein sequences can be obtained from the ORCAE database (https://bioinformatics.psb.ugent.be/orcae/overview/Tetur) [30] using the tetur IDs provided in the spider mite DOG nomenclature section, while DOG protein sequences of *Panonychus ulmi* and *Panonychus**citri* were obtained from Bajda et al. 2015 [31]. DOG protein sequences of other Tetranychid species were derived from Matsuda et al. [32] and can be found in File S1. *Brevipalpus yothersi* DOG protein sequences can be obtained from https://bioinformatics.psb.ugent.be/orcae/overview/Bryot [34] using the bryot IDs provided in Fig. 1.

## Abbreviations

β-KAP: Beta ketoadipate pathway
CM: Castlemart
CPM: Counts per million
CYPs: Cytochrome P450 mono-oxygenases
DOG: Intradiol ring cleavage dioxygenases
OCR: Oxygen consumption rate
ORCAE: Online resource for community annotation of eukaryotes
UPLC-MS: Ultra performance liquid chromatography-mass spectrometer

## References

[1] Neish AC (1960). Biosynthetic pathways of aromatic compounds1•2.

[2] Dao TTH, Linthorst HJM, Verpoorte R (2011). Chalcone synthase and its functions in plant resistance. Phytochem Rev..

[3] Mazid, Ta K, Mohammad F. Role of secondary metabolites in defense mechanisms of plants. 2011. www.biolmedonline.com. Accessed 19 Jan 2021.

[4] Guzik U, Hupert-kocurek K (2013). Intradiol dioxygenases — the key enzymes in xenobiotics degradation. Biodegrad Hazard Spec Prod.

[5] Bugg TDH, Winfield CJ (1998). Enzymatic cleavage of aromatic rings: mechanistic aspects of the catechol dioxygenases and later enzymes of bacterial oxidative cleavage pathways. Nat Prod Rep..

[6] Wells T, Ragauskas AJ (2012). Biotechnological opportunities with the β-ketoadipate pathway. Trends Biotechnol.

[7] MacLean AM, MacPherson G, Aneja P, Finan TM (2006). Characterization of the β-ketoadipate pathway in Sinorhizobium meliloti. Appl Environ Microbiol..

[8] Cha CJ (2006). Catechol 1,2-Dioxygenase from *Rhodococcus rhodochrous* N75 capable of metabolizing alkyl-substituted catechols. J Microbiol Biotechnol..

[9] Tsai SC, Li YK (2007). Purification and characterization of a catechol 1,2-dioxygenase from a phenol degrading *Candida albicans* TL3. Arch Microbiol..

[10] Harayamas S, Rekik M (1989). The journal of biological chemlstry bacterial aromatic ring-cleavage enzymes are classified into two different gene families*.

[11] Sherr CJ (1994). The ins and outs of ring cleavage dioxygenases. Trends Cell Biol..

[12] Fetzner S (2012). Ring-cleaving dioxygenases with a cupin fold. Appl Environ Microbiol..

[13] Grbić M, Van Leeuwen T, Clark RM, Rombauts S, Rouzé P, Grbić V, Osborne EJ, Dermauw W, Ngoc T, Cao P, Ortego F, Hernández-Crespo P, Diaz I, Martinez M, Navajas M, Sucena É, Magalhães S, Nagy L, Pace RM, Djuranović Y (2011). The genome of *Tetranychus urticae* reveals herbivorous pest adaptations. Nature..

[14] Dermauw W, Wybouw N, Rombauts S, Menten B, Vontas J, Grbi M (2013). A link between host plant adaptation and pesticide resistance in the polyphagous spider mite *Tetranychus urticae*. Proc Natl Acad Sci U S A..

[15] Wybouw N, Van Leeuwen T, Dermauw W (2018). A massive incorporation of microbial genes into the genome of *Tetranychus urticae*, a polyphagous arthropod herbivore. Insect Mol Biol..

[16] Schlachter CR, Daneshian L, Amaya J, Klapper V, Wybouw N, Borowski T (2019). Structural and functional characterization of an intradiol ring-cleavage dioxygenase from the polyphagous spider mite herbivore *Tetranychus urticae Koch*. Insect Biochem Mol Biol..

[17] Ferraroni M, Kolomytseva MP, Solyanikova IP, Scozzafava A, Golovleva LA, Briganti F (2006). Crystal structure of 3-chlorocatechol 1,2-dioxygenase key enzyme of a new modified ortho-pathway from the gram-positive *Rhodococcus opacus* 1CP grown on 2-chlorophenol. J Mol Biol..

[18] Matera I, Ferraroni M, Kolomytseva M, Golovleva L, Scozzafava A, Briganti F (2010). Catechol 1,2-dioxygenase from the Gram-positive *Rhodococcus opacus* 1CP: quantitative structure/activity relationship and the crystal structures of native enzyme and catechols adducts. J Struct Biol..

[19] Bianchetti CM, Harmann CH, Takasuka TE, Hura GL, Dyer K, Fox BG (2013). Fusion of dioxygenase and lignin-binding domains in a novel secreted enzyme from cellulolytic streptomyces sp. SIRexaa-e. J Biol Chem.

[20] Bennett RN, Wallsgrove RM (1994). Secondary metabolites in plant defence mechanisms. New Phytol..

[21] Erb M, Kliebenstein DJ (2020). Plant secondary metabolites as defenses, regulators, and primary metabolites: the blurred functional trichotomy. Plant Physiol..

[22] Snoeck S, Wybouw N, Van LT, Dermauw W (2018). Transcriptomic plasticity in the arthropod generalist *Tetranychus urticae* upon long-term acclimation to different host plants. G3.

[23] Wybouw N, Zhurov V, Martel C, Bruinsma KA, Hendrickx F, Grbic V (2015). Adaptation of a polyphagous herbivore to a novel host plant extensively shapes the transcriptome of herbivore and host. Mol Ecol..

[24] Roopesh K, Guyot S, Sabu A, Haridas M, Isabelle PG, Roussos S (2010). Biotransformation of procyanidins by a purified fungal dioxygenase: identification and characterization of the products using mass spectrometry. Process Biochem..

[25] Van Pottelberge S, Van Leeuwen T, Nauen R, Tirry L (2009). Resistance mechanisms to mitochondrial electron transport inhibitors in a field-collected strain of *Tetranychus urticae Koch* (Acari: Tetranychidae). Bull Entomol Res..

[26] Kant MR, Ament K, Sabelis MW, Haring MA, Schuurink RC (2004). Differential timing of spider mite-induced direct and indirect defenses in tomato plants. Plant Physiol..

[27] Kant MR, Sabelis MW, Haring MA, Schuurink RC (2008). Intraspecific variation in a generalist herbivore accounts for differential induction and impact of host plant defences. Proc R Soc B Biol Sci..

[28] Alba JM, Schimmel BCJ, Glas JJ, Ataide LMS, Pappas ML, Villarroel CA (2015). Spider mites suppress tomato defenses downstream of jasmonate and salicylate independently of hormonal crosstalk. New Phytol..

[29] Alavijeh ES, Khajehali J, Snoeck S, Panteleri R, Ghadamyari M, Jonckheere W (2020). Molecular and genetic analysis of resistance to METI-I acaricides in Iranian populations of the citrus red mite *Panonychus citri*. Pestic Biochem Physiol..

[30] Sterck L, Billiau K, Abeel T, Rouzé P, Van de Peer Y. ORCAE: online resource for community annotation of eukaryotes. Nat Methods. (https://bioinformatics.psb.ugent.be/orcae/overview/Tetur. 2012. 10.1038/nmeth.2242. 10.1038/nmeth.2242 23132114

[31] Bajda S, Dermauw W, Greenhalgh R, Nauen R, Tirry L, Clark RM (2015). Transcriptome profiling of a spirodiclofen susceptible and resistant strain of the European red mite *Panonychus ulmi* using strand-specific RNA-seq. BMC Genom..

[32] Matsuda T, Kozaki T, Ishii K, Gotoh T (2018). Phylogeny of the spider mite sub-family Tetranychinae (Acari: Tetranychidae) inferred from RNA-Seq data. PLoS One..

[33] Rice P, Longden L, Bleasby A (2000). EMBOSS: the european molecular biology open software suite. Trends Genet.

[34] Navia D, Novelli VM, Rombauts S, Freitas-Astúa J, Santos de Mendonça R, Nunes MA, et al. Draft genome assembly of the false spider mite *Brevipalpus yothersi*. Microbiol Resour Announc. 2019;8. 10.1128/mra.01563-18https://bioinformatics.psb.ugent.be/orcae/overview/Bryot. 10.1128/MRA.01563-18 PMC6368659 30746524

[35] Li W, Godzik A (2006). Cd-hit: A fast program for clustering and comparing large sets of protein or nucleotide sequences. Bioinformatics..

[36] Katoh K, Standley DM (2013). MAFFT multiple sequence alignment software version 7: improvements in performance and usability. Mol Biol Evol..

[37] Trifinopoulos J, Nguyen LT, von Haeseler A, Minh BQ (2016). W-IQ-TREE: a fast online phylogenetic tool for maximum likelihood analysis. Nucleic Acids Res..

[38] Stamatakis A (2014). RAxML version 8: a tool for phylogenetic analysis and post-analysis of large phylogenies. Bioinformatics..

[39] Miller MA, Pfeiffer W, Schwartz T. Creating the CIPRES Science Gateway for inference of large phylogenetic trees. 2010 Gateway Computing Environments Workshop (GCE). 2010;0:1–8. 10.1109/GCE.2010.5676129.

[40] Moore RM, Harrison AO, McAllister SM, Polson SW, Wommack KE (2020). Iroki: automatic customization and visualization of phylogenetic trees. PeerJ..

[41] Tamura K, Stecher G, Peterson D, Filipski A, Kumar S (2013). MEGA6: Molecular evolutionary genetics analysis version 6.0. Mol Biol Evol..

[42] Demaeght P, Dermauw W, Tsakireli D, Khajehali J, Nauen R, Tirry L (2013). Molecular analysis of resistance to acaricidal spirocyclic tetronic acids in *Tetranychus urticae*: CYP392E10 metabolizes spirodiclofen, but not its corresponding enol. Insect Biochem Mol Biol..

[43] Wybouw N, Dermauw W, Tirry L, Stevens C, Grbic M, Feyereisen R (2014). A gene horizontally transferred from bacteria protects arthropods from host plant cyanide poisoning. Elife..

[44] Khalighi M, Dermauw W, Wybouw N, Bajda S, Osakabe M, Tirry L (2015). Molecular analysis of cyenopyrafen resistance in the two-spotted spider mite *Tetranychus urticae*. Pest Manag Sci..

[45] Wybouw N (2015). The role of horizontally transferred genes in the xenobiotic adaptations of the spider mite *Tetranychus urticae*.

[46] Jonckheere W, Dermauw W, Zhurov V, Wybouw N, Van Den Bulcke J, Villarroel CA (2016). The salivary protein repertoire of the polyphagous spider mite *Tetranychus urticae*: a quest for effectors. Mol Cell Proteomics..

[47] Pavlidi N, Khalighi M, Myridakis A, Dermauw W, Wybouw N, Tsakireli D, et al. A glutathione-S-transferase (TuGSTd05) associated with acaricide resistance in *Tetranychus urticae* directly metabolizes the complex II inhibitor cyflumetofen. Insect Biochem Mol Biol. 2017. 10.1016/j.ibmb.2016.12.003 27932274

[48] Snoeck S, Pavlidi N, Pipini D, Vontas J, Dermauw W, Van Leeuwen T. Substrate specificity and promiscuity of horizontally transferred UDP-glycosyltransferases in the generalist herbivore *Tetranychus urticae*. Insect Biochem Mol Biol. 2019. 10.1016/J.IBMB.2019.04.010. 10.1016/j.ibmb.2019.04.010 30978500

[49] Bryon A, Wybouw N, Dermauw W, Tirry L, Van Leeuwen T (2013). Genome wide gene-expression analysis of facultative reproductive diapause in the two-spotted spider mite *Tetranychus urticae*. BMC Genom.

[50] Gu Z, Eils R, Schlesner M (2016). Complex heatmaps reveal patterns and correlations in multidimensional genomic data. Bioinformatics..

[51] Ngoc PCT, Greenhalgh R, Dermauw W, Rombauts S, Bajda S, Zhurov V (2016). Complex evolutionary dynamics of massively expanded chemosensory receptor families in an extreme generalist chelicerate herbivore. Genome Biol Evol..

[52] Anders S, Pyl PT, Huber W (2015). HTSeq-A Python framework to work with high-throughput sequencing data. Bioinformatics..

[53] Love MI, Huber W, Anders S (2014). Moderated estimation of fold change and dispersion for RNA-seq data with DESeq2. Genome Biol..

[54] Robinson MD, McCarthy DJ, Smyth GK (2010). edgeR: a Bioconductor package for differential expression analysis of digital gene expression data. Bioinformatics..

[55] Livak KJ, Schmittgen TD (2001). Analysis of relative gene expression data using real-time quantitative PCR and the 2−ΔΔCT method. Methods..

[56] Yang C, Pan H, Liu Y, Zhou X (2015). Stably expressed housekeeping genes across developmental stages in the two-spotted spider mite, *Tetranychus urticae*. PLoS One.

[57] Untergasser A, Cutcutache I, Koressaar T, Ye J, Faircloth BC, Remm M (2012). Primer3—new capabilities and interfaces. Nucleic Acids Res..

[58] Froger A, Hall JE. Transformation of Plasmid DNA into E. Coli using the heat shock method. J Vis Exp. 2007;6. 10.3791/253. 10.3791/253 PMC2557105 18997900

[59] Fisher C (2019). Phenol-chloroform extraction for dsRNA purification.

[60] Dermauw W, Jonckheere W, Riga M, Livadaras I, Vontas J, Van Leeuwen T (2020). Targeted mutagenesis using CRISPR-Cas9 in the chelicerate herbivore *Tetranychus urticae*. Insect Biochem Mol Biol..

[61] Almagro Armenteros JJ, Tsirigos KD, Sønderby CK, Petersen TN, Winther O, Brunak S (2019). SignalP 5.0 improves signal peptide predictions using deep neural networks. Nat Biotechnol..

[62] Ring H, Gao Z, Klein ND, Garwood M, Bischof JC, Haynes CL (2018). Ferrozine assay for simple and cheap iron analysis of silica-coated iron oxide nanoparticles: ferrozine assay for simple and cheap iron analysis of silica-coated iron oxide.

[63] Kaulmann U, Kaschabek SR, Schlomann M (2001). Mechanism of chloride elimination from 3-chloro-and 2,4-dichloro-cis,cis-muconate: new insight obtained from analysis of muconate cycloisomerase variant CatB-K169A. J Bacteriol..

[64] Vanholme R, Sundin L, Seetso KC, Kim H, Liu X, Li J (2019). COSY catalyses trans–cis isomerization and lactonization in the biosynthesis of coumarins. Nat Plants..

[65] Migeon A, Nouguier E, Dorkeld F (2010). Spider Mites Web: A comprehensive database for the Tetranychidae.

[66] Alberti G, Crooker A, Helle W, Sabelis MW (1985). Internal anatomy. Spider mites: their biology, natural enemies and control.

[67] Semana P, Powlowski J. Four aromatic intradiol ring cleavage dioxygenases from *Aspergillus niger*. 2019;85:1–18. 10.1128/AEM.01786-19 PMC6856329 31540981

[68] Kumar A, Trefault N, Olaniran AO (2016). Microbial degradation of 2,4-dichlorophenoxyacetic acid: insight into the enzymes and catabolic genes involved, their regulation and biotechnological implications. Crit Rev Microbiol..

[69] Pimviriyakul P, Wongnate T, Tinikul R, Chaiyen P (2020). Microbial degradation of halogenated aromatics: molecular mechanisms and enzymatic reactions. Microb Biotechnol..

[70] Swetha VP, Phale PS (2005). Metabolism of carbaryl via 1,2-dihydroxynaphthalene by soil isolates *Pseudomonas sp.* strains C4, C5, and C6. Appl Environ Microbiol..

[71] Howe GA, Jander G (2008). Plant immunity to insect herbivores. Annu Rev Plant Biol..

[72] Ehrlich PR, Raven PH (1964). Butterflies and plants: a study in coevolution..

[73] Poulton JE (1990). Cyanogenesis in plants1. Plant Physiol..

[74] Halkier BA, Gershenzon J (2006). Biology and biochemistry of glucosinolates. Annu Rev Plant Biol..

[75] Li X, Schuler MA, Berenbaum MR (2007). Molecular mechanisms of metabolic resistance to synthetic and natural xenobiotics. Annu Rev Entomol..

[76] Van Leeuwen T, Dermauw W (2016). The molecular evolution of xenobiotic metabolism and resistance in chelicerate mites.

[77] Feyereisen R, Dermauw W, Van Leeuwen T (2015). Genotype to phenotype, the molecular and physiological dimensions of resistance in arthropods. Pestic Biochem Physiol..

[78] Bensoussan N, Zhurov V, Yamakawa S, O’Neil CH, Suzuki T, Grbić M (2018). The digestive system of the two-spotted spider mite, *Tetranychus urticae Koch*, in the context of the mite-plant interaction. Front Plant Sci..

[79] Ahn S-J, Dermauw W, Wybouw N, Heckel DG, Van Leeuwen T (2014). Bacterial origin of a diverse family of UDP-glycosyltransferase genes in the *Tetranychus urticae* genome. Insect Biochem Mol Biol..

[80] Sterkel M, Oliveira PL (2017). Developmental roles of tyrosine metabolism enzymes in the blood-sucking insect *Rhodnius prolixus*.

[81] Itoh Y, Shimotsuma Y, Jouraku A, Dermauw W, Van Leeuwen T, Osakabe M. Combination of target site mutation and associated CYPs confers high-level resistance to pyridaben in *Tetranychus urticae*. Pestic Biochem Physiol. 2021;105000. 10.1016/J.PESTBP.2021.105000. 10.1016/j.pestbp.2021.105000 35082027

[82] Bryon A, Kurlovs AH, Dermauw W, Greenhalgh R, Riga M, Grbic M (2017). Disruption of a horizontally transferred phytoene desaturase abolishes carotenoid accumulation and diapause in *Tetranychus urticae*. Proc Natl Acad Sci U S A..

[83] Von Lintig J, Wyss A (2001). Molecular analysis of vitamin A formation: cloning and characterization of β-carotene 15,15′-dioxygenases. Arch Biochem Biophys..

[84] Ahrazem O, Gómez-Gómez L, Rodrigo MJ, Avalos J, Limón MC (2016). Carotenoid cleavage oxygenases from microbes and photosynthetic organisms: features and functions. Int J Mol Sci..

[85] Meng N, Wei Y, Gao Y, Yu K, Cheng J, Li X-Y (2020). Characterization of transcriptional expression and regulation of carotenoid cleavage dioxygenase 4b in grapes. Front Plant Sci..

[86] Kortbeek RWJ, van der Gragt M, Bleeker PM (2019). Endogenous plant metabolites against insects. Eur J Plant Pathol..

[87] Misra P, Pandey A, Tiwari M, Chandrashekar K, Sidhu OP, Asif MH (2010). Modulation of transcriptome and metabolome of tobacco by Arabidopsis transcription factor, AtMYB12, leads to insect resistance. Plant Physiol..

[88] Mohammed AlJabr A, Hussain A, Rizwan-ul-Haq M, Al-Ayedh H (2017). Toxicity of plant secondary metabolites modulating detoxification genes expression for natural red palm weevil pesticide development. Molecules..

[89] Movva V, Pathipati UR (2017). Feeding-induced phenol production in Capsicum annuum L. influences *Spodoptera litura F.* larval growth and physiology. Arch Insect Biochem Physiol..

[90] Onkokesung N, Reichelt M, Van Doorn A, Schuurink RC, Van Loon JJA, Dicke M (2014). Modulation of flavonoid metabolites in Arabidopsis thaliana through overexpression of the MYB75 transcription factor: role of kaempferol-3,7- dirhamnoside in resistance to the specialist insect herbivore *Pieris brassicae*. J Exp Bot..

[91] Liu Q, Gao T, Liu W, Liu Y, Zhao Y, Liu Y (2020). Functions of dopamine in plants: a review. Plant Signal Behav..

[92] Kielkiewicz M (2002). Influence of carmine spider mite *Tetranychus cinnabarinus Boisd.* (Acarida: Tetranychidae) feeding on ethylene production and the activity of oxidative enzymes in damaged tomato plants. Acarid Phylogeny and Evolution: Adaptation in Mites and Ticks.

[93] Kundu A, Vadassery J (2019). Chlorogenic acid-mediated chemical defence of plants against insect herbivores. Plant Biol..

[94] Wadke N, Kandasamy D, Vogel H, Lah L, Wingfield BD, Paetz C (2016). The Bark-Beetle-associated fungus, *Endoconidiophora polonica*, utilizes the phenolic defense compounds of its host as a carbon source. Plant Physiol..

[95] Wybouw N, Pauchet Y, Heckel DG, Van LT (2016). Horizontal gene transfer contributes to the evolution of arthropod herbivory. Genome Biol Evol..

[96] Bensoussan N, Santamaria ME, Zhurov V, Diaz I, Grbić M, Grbić V (2016). Plant-herbivore interaction: dissection of the cellular pattern of *Tetranychus urticae* feeding on the host plant. Front Plant Sci..

[97] Denness L, McKenna JF, Segonzac C, Wormit A, Madhou P, Bennett M (2011). Cell wall damage-induced lignin biosynthesis is regulated by a reactive oxygen species- and jasmonic acid-dependent process in arabidopsis. Plant Physiol..

[98] Moura JCMS, Bonine CAV, de Oliveira Fernandes Viana J, Dornelas MC, Mazzafera P. (2010). Abiotic and biotic stresses and changes in the lignin content and composition in plants. J Integr Plant Biol..

[99] Xie M, Zhang J, Tschaplinski TJ, Tuskan GA, Chen J-G, Muchero W (2018). Regulation of lignin biosynthesis and its role in growth-defense tradeoffs. Front Plant Sci..

[100] Duffey SS, Stout MJ (1996). Antinutritive and toxic components of plant defense against insects. Arch Insect Biochem Physiol..

[101] Constabel CP, Barbehenn R. Defensive roles of polyphenol oxidase in plants. In: Schaller A, editor. Induced plant resistance to herbivory. Dordrecht: Springer; 2008. p. 253–70. 10.1007/978-1-4020-8182-8_12.

[102] Martel C, Zhurov V, Navarro M, Martinez M, Cazaux M, Auger P (2015). Tomato whole genome transcriptional response to *Tetranychus urticae* identifies divergence of spider mite-induced responses between tomato and arabidopsis. Mol Plant Microbe Interact.

[103] Howe GA, Ryan CA (1999). Suppressors of systemin signaling identify genes in the tomato wound response pathway.

[104] Richter C, Dirks ME, Gronover CS, Prüfer D, Moerschbacher BM (2012). Silencing and heterologous expression of ppo-2 indicate a specific function of a single polyphenol oxidase isoform in resistance of dandelion ( Taraxacum officinale ) against Pseudomonas syringae pv. Tomato.

[105] Rao MJ, Xu Y, Huang Y, Tang X, Deng X, Xu Q (2019). Ectopic expression of citrus UDP-GLUCOSYL TRANSFERASE gene enhances anthocyanin and proanthocyanidins contents and confers high light tolerance in Arabidopsis. BMC Plant Biol..

[106] Dixon RA, Xie D-Y, Sharma SB (2004). Proanthocyanidins - a final frontier in flavonoid research?. New Phytol..

[107] Ferraroni M, Seifert J, Travkin VM, Thiel M, Kaschabek S, Scozzafava A (2005). Crystal structure of the hydroxyquinol 1,2-dioxygenase from Nocardioides simplex 3E, a key enzyme involved in polychlorinated aromatics biodegradation. J Biol Chem..

[108] Orville AM, Lipscomb JD, Ohlendorf DH (1997). Crystal structures of substrate and substrate analog complexes of protocatechuate 3,4-dioxygenase: Endogenous Fe3+ ligand displacement in response to substrate binding. Biochemistry..

[109] Vetting MW, Ohlendorf DH (2000). The 1.8 Å crystal structure of catechol 1,2-dioxygenase reveals a novel hydrophobic helical zipper as a subunit linker. Structure..

[110] Van Leeuwen T, Demaeght P, Osborne E, Dermauw W, Gohlke S, Nauen R (2012). Population bulk segregant mapping uncovers resistance mutations and the mode of action of a chitin synthesis inhibitor in arthropods. Proc Natl Acad Sci U S A..

