# Supplementary material for: Intradiol ring cleavage dioxygenases from herbivorous spider mites as a new detoxification enzyme family in animals
Source: BMC Biol. 2022 Jun 4;20:131. doi: 10.1186/s12915-022-01323-1 (PMC9167512; doi:10.1186/s12915-022-01323-1)
Supplement: Supplementary file 4 — Additional file 4: File S3. Coverage plots of 17 T. urticae DOG genes and their surrounding regions in the genome of Tetranychus urticae. Gene models of DOG genes and their neighboring genes are depicted as follows: large and small orange boxes represent coding sequences and untranslated regions, respectively, whereas introns are shown as connecting lines between the boxes. (+) and (-) represent the forward and reverse strand, respectively. Underneath the gene models of TuDOG7, TuDOG10 and TuDOG11, the length and position of an amplicon obtained by PCR (Fig. S4) is indicated with a red line. Next, coverage plots of Illumina-reads (‘“Illu.’”) from adult T. urticae polyA selected RNA [13] and from genomic DNA sequencing of the EtoxR (‘“Etox’”) and London(‘“Lon’”) strain of T. urticae [13, 110] are shown below the gene models. The Illumina reads coverage plots are followed by a coverage plot of Sanger (‘“San.’”) reads from genomic DNA sequencing of the London strain [13] and by an alignment of these Sanger reads with the T. urticae genome of the London strain. Paired-end Sanger reads for which both reads are mapped in or extend nearby the indicated region are denoted by thin lines to show pair connections [13] (see [43], for mapping details). [file 12915_2022_1323_MOESM4_ESM.pdf]

TuDOG1 (*tetur01g00490*, pseudochromosome_2)

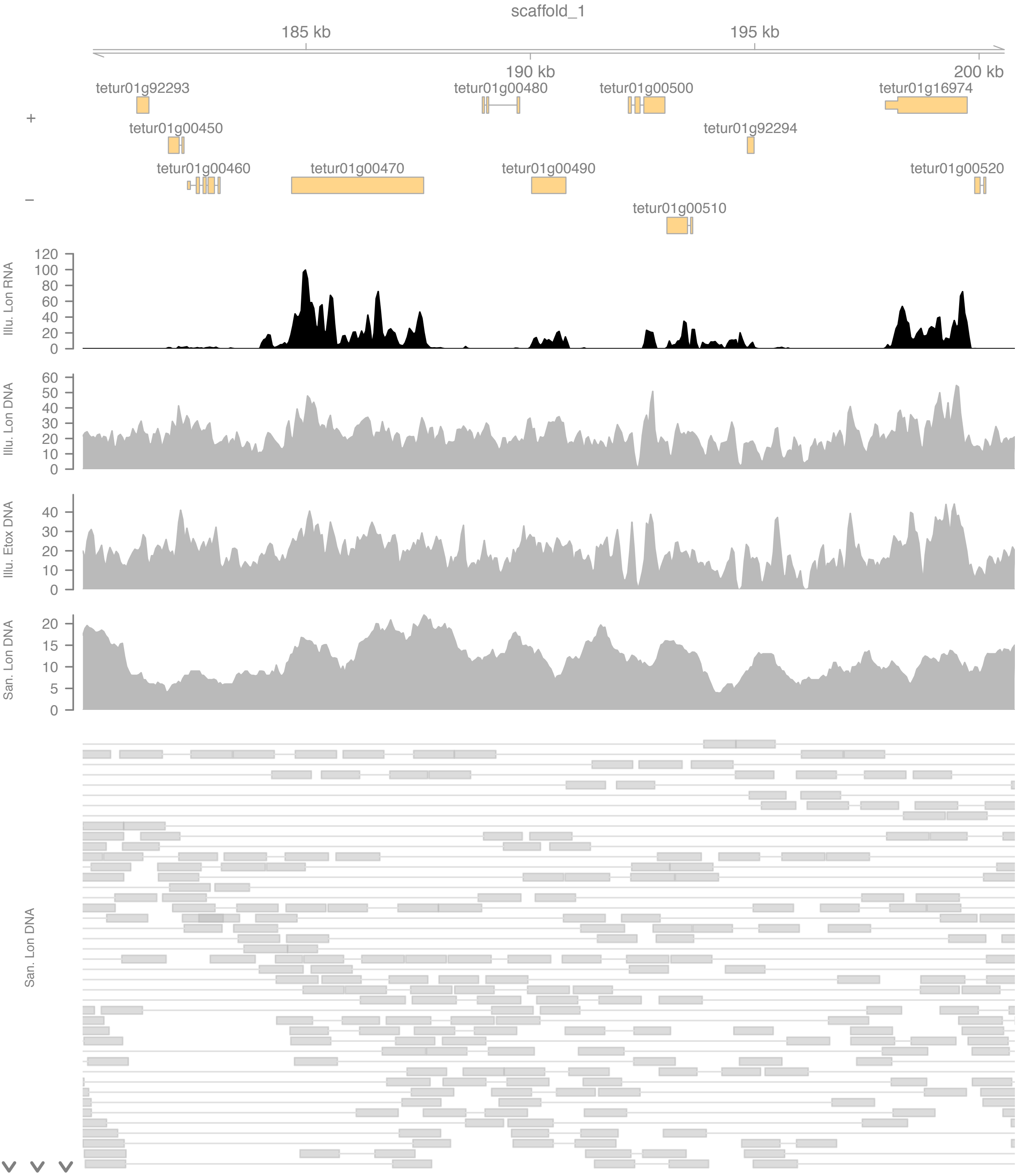

TuDOG2 (*tetur04g00150*, pseudochromosome_3)

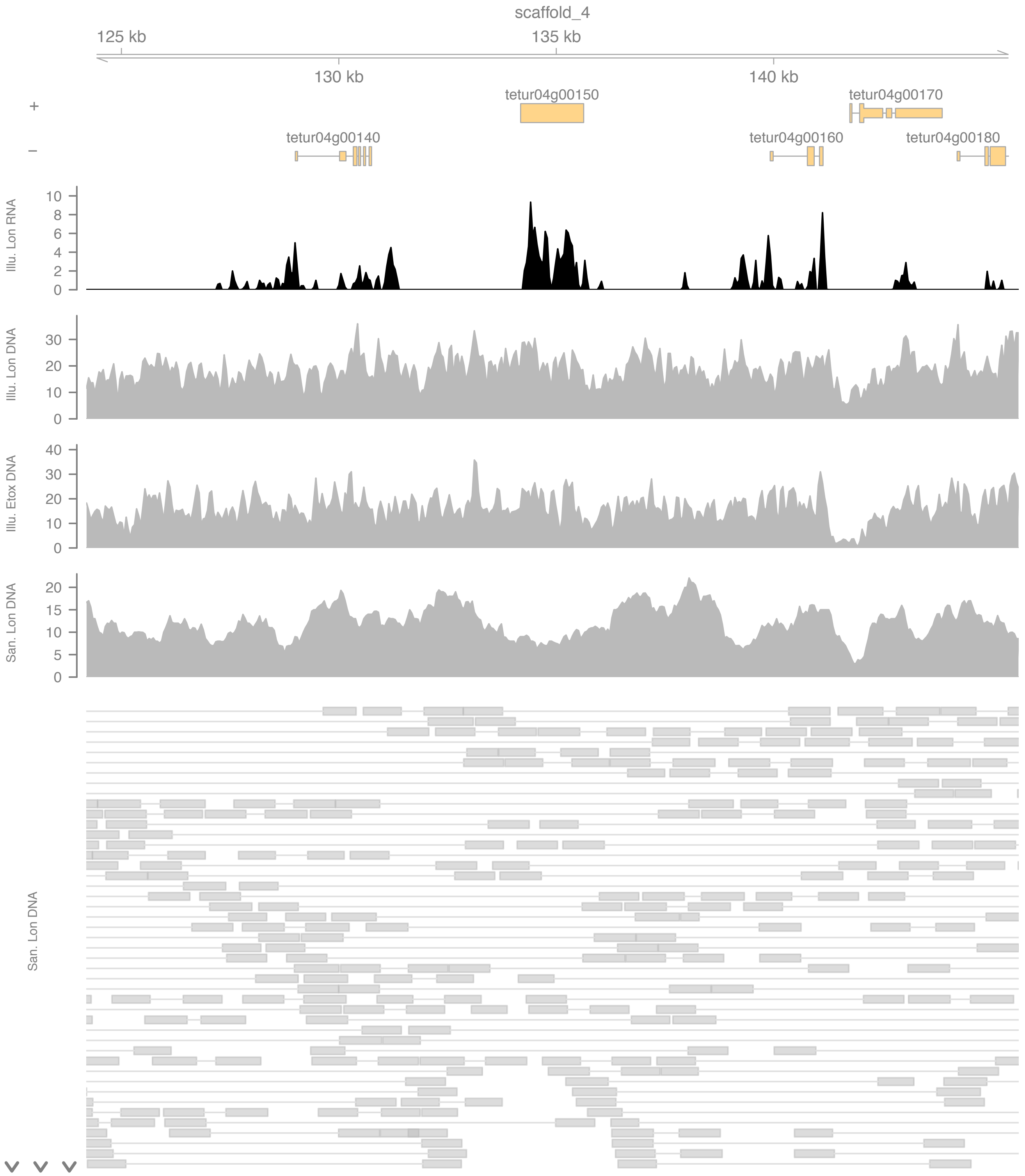

TuDOG3 (*tetur04g08620*, pseudochromosome_2)

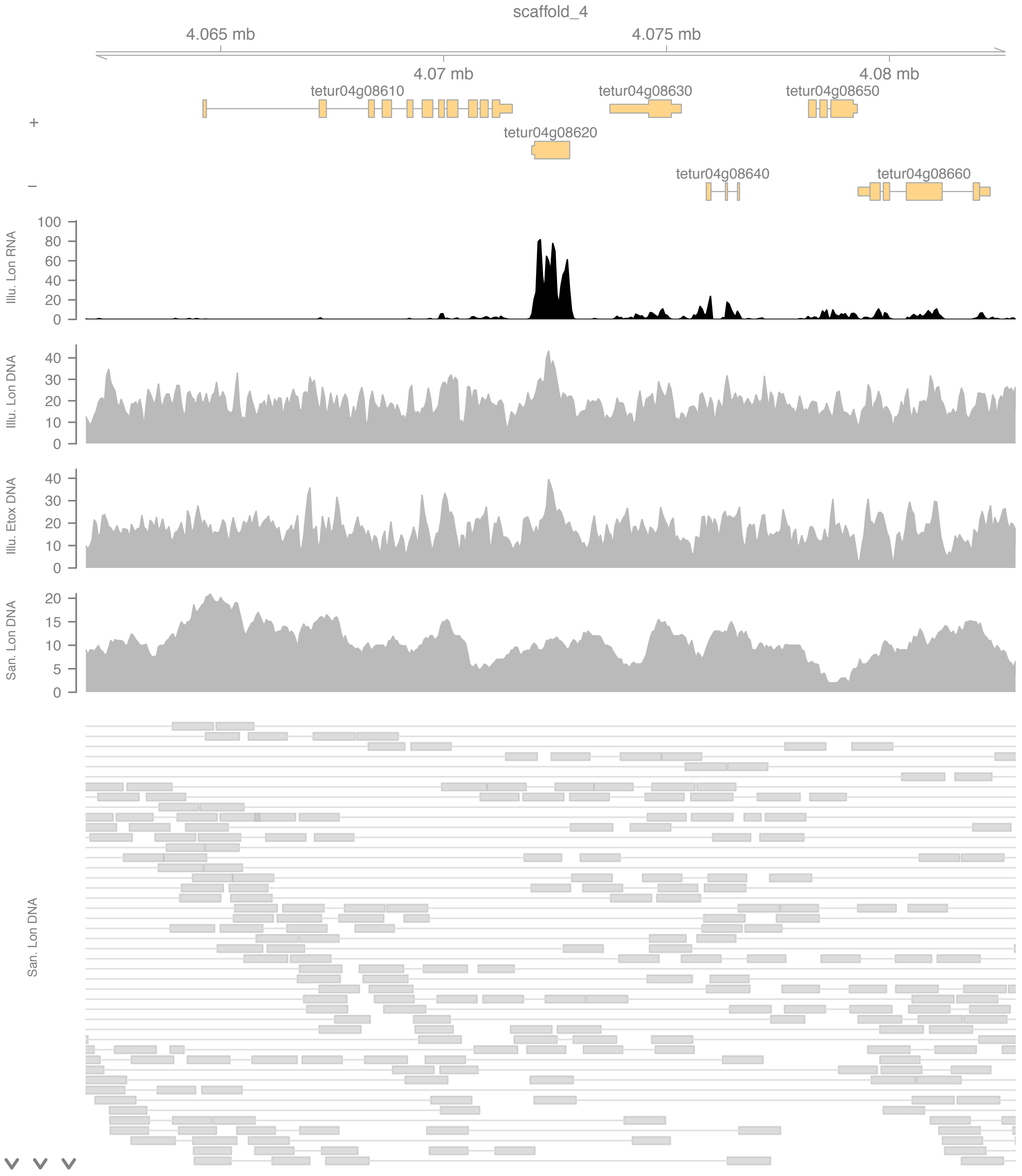

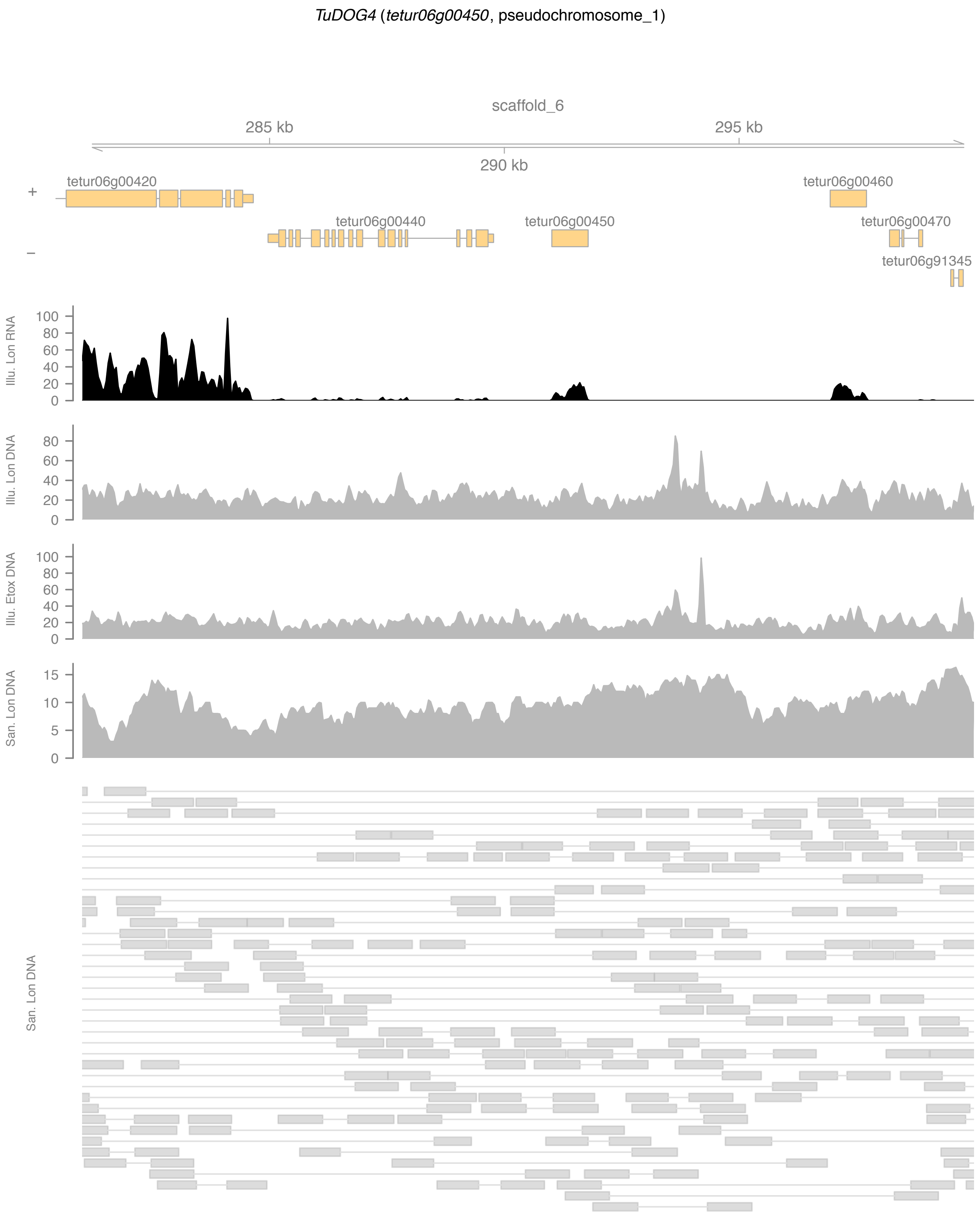

TuDOG4 (*tetur06g00450*, pseudochromosome_1)
scaffold_6
285 kb
290 kb
295 kb
+
–
tetur06g00420
tetur06g00440
tetur06g00450
tetur06g00460
tetur06g00470
tetur06g91345
Illu. Lon RNA
Illu. Lon DNA
Illu. Etox DNA
San. Lon DNA
San. Lon DNA

TuDOG5 (*tetur06g00460*, pseudochromosome_1)

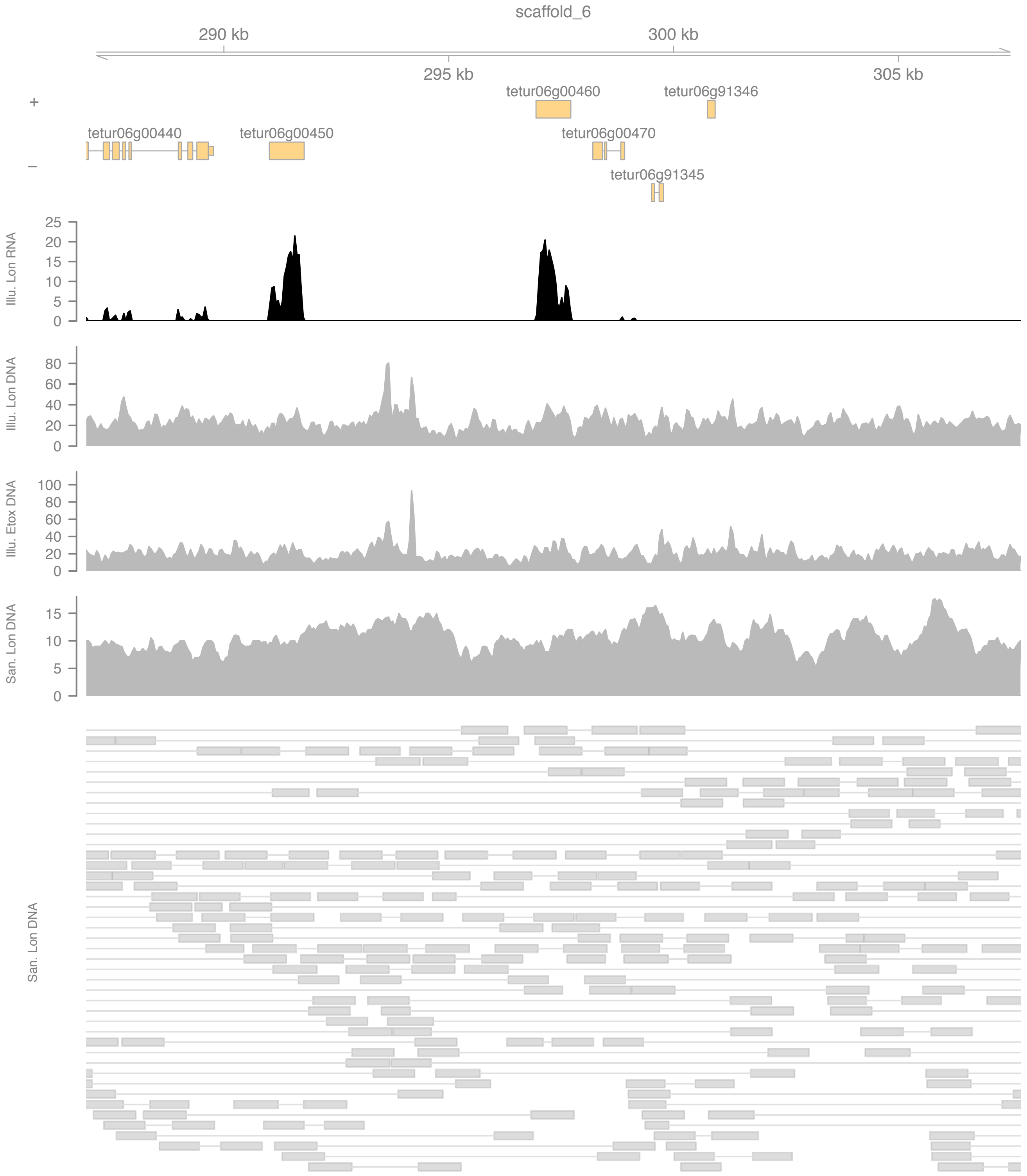

TuDOG6 (*tetur07g02040*, pseudochromosome_1)

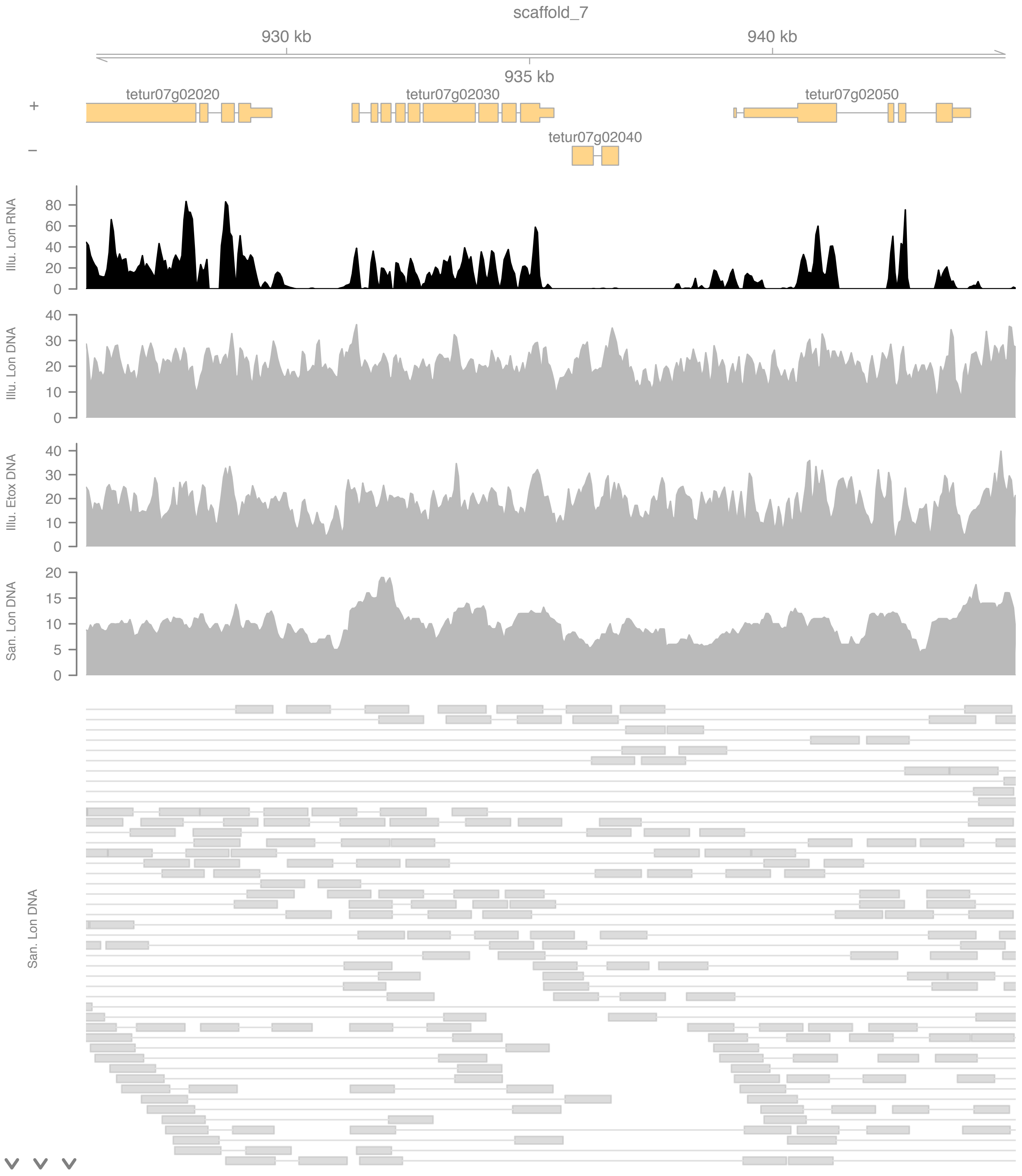

TuDOG7 (*tetur07g05930*, pseudochromosome_1)

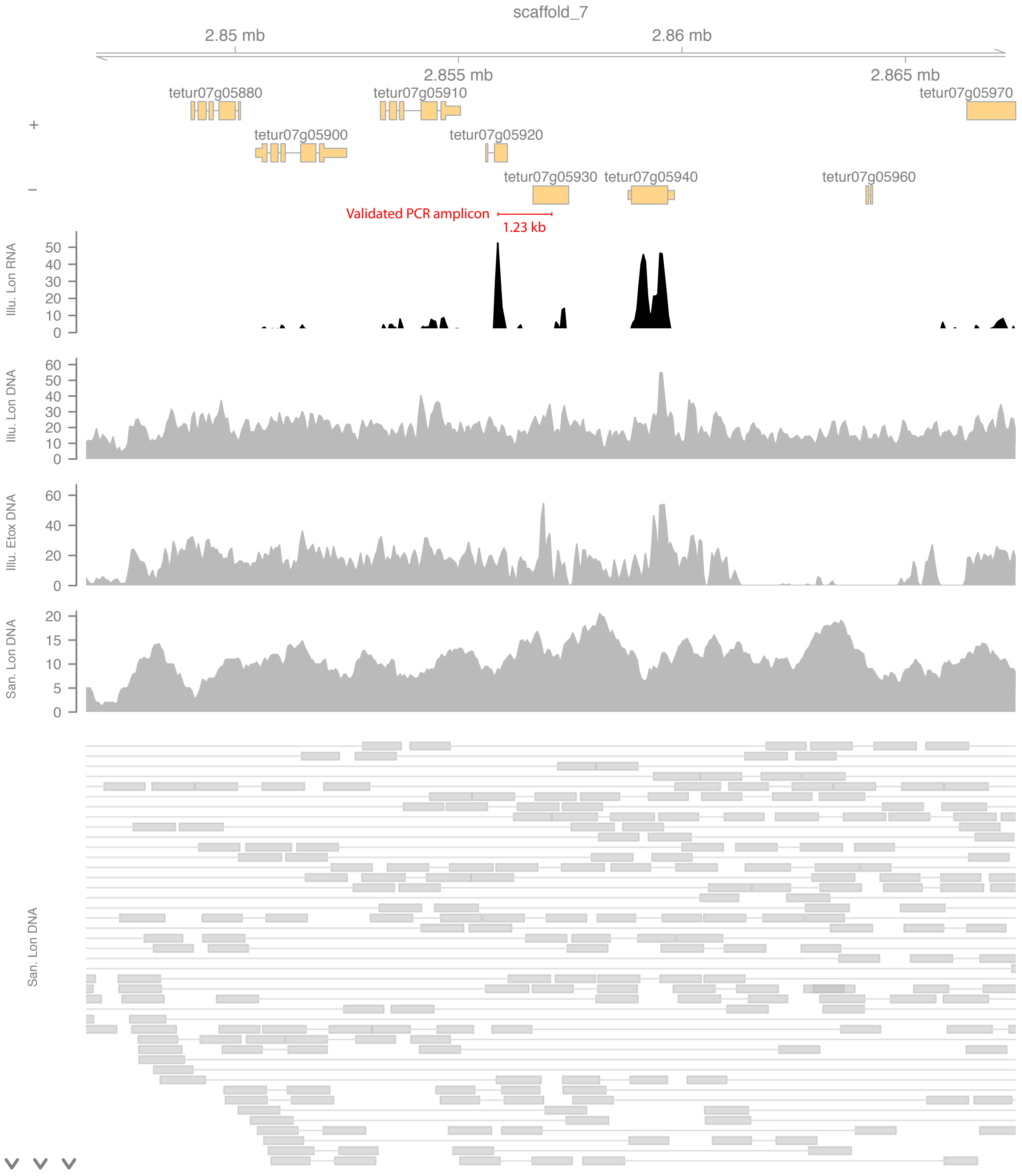

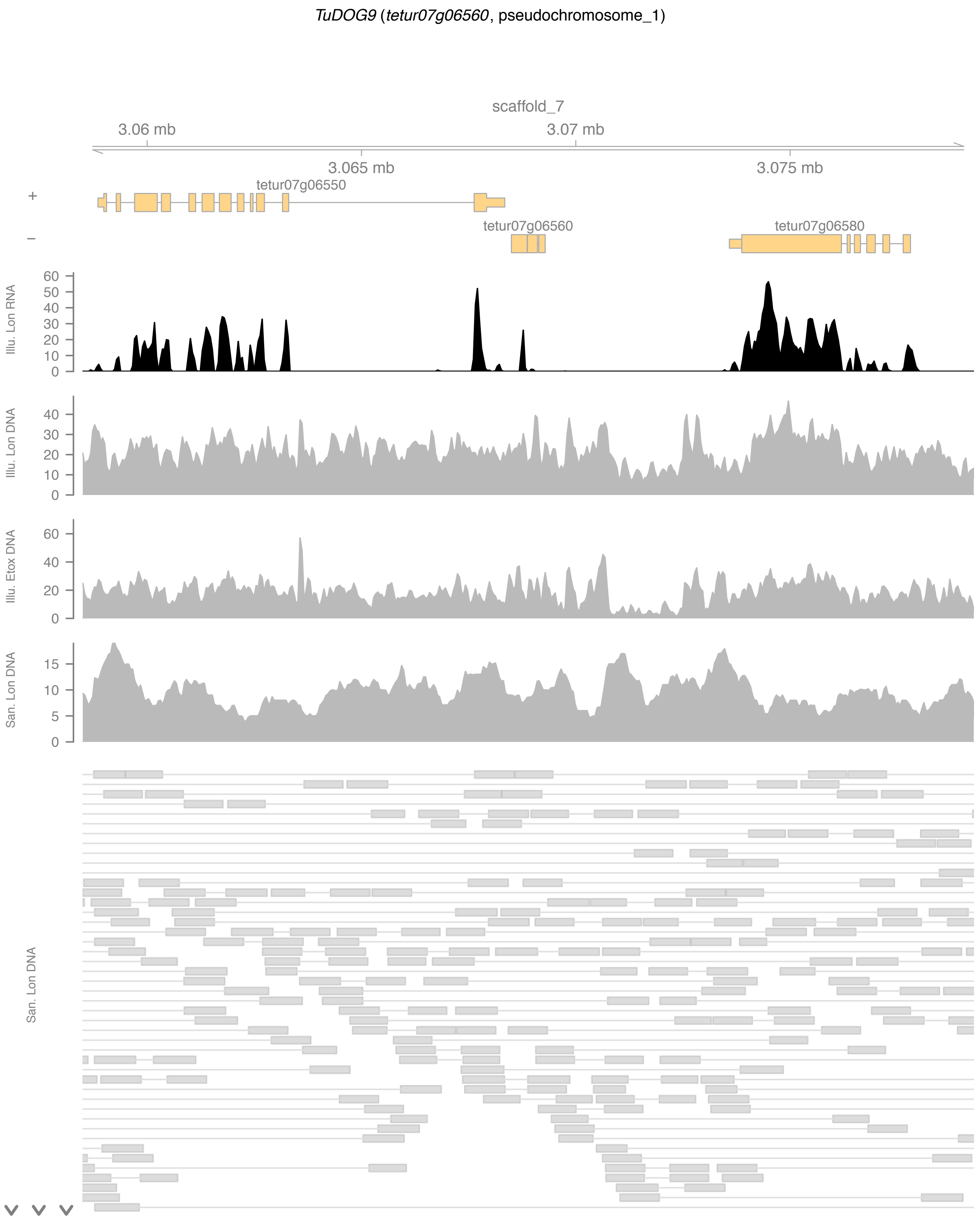
TuDOG9 (tetur07g06560, pseudochromosome_1)
scaffold_7
3.06 mb
3.065 mb
3.07 mb
3.075 mb
+
−
tetur07g06550
tetur07g06560
tetur07g06580
Illu. Lon RNA
Illu. Lon DNA
Illu. Etox DNA
San. Lon DNA
San. Lon DNA

TuDOG10 (*tetur12g04671*, pseudochromosome_1)

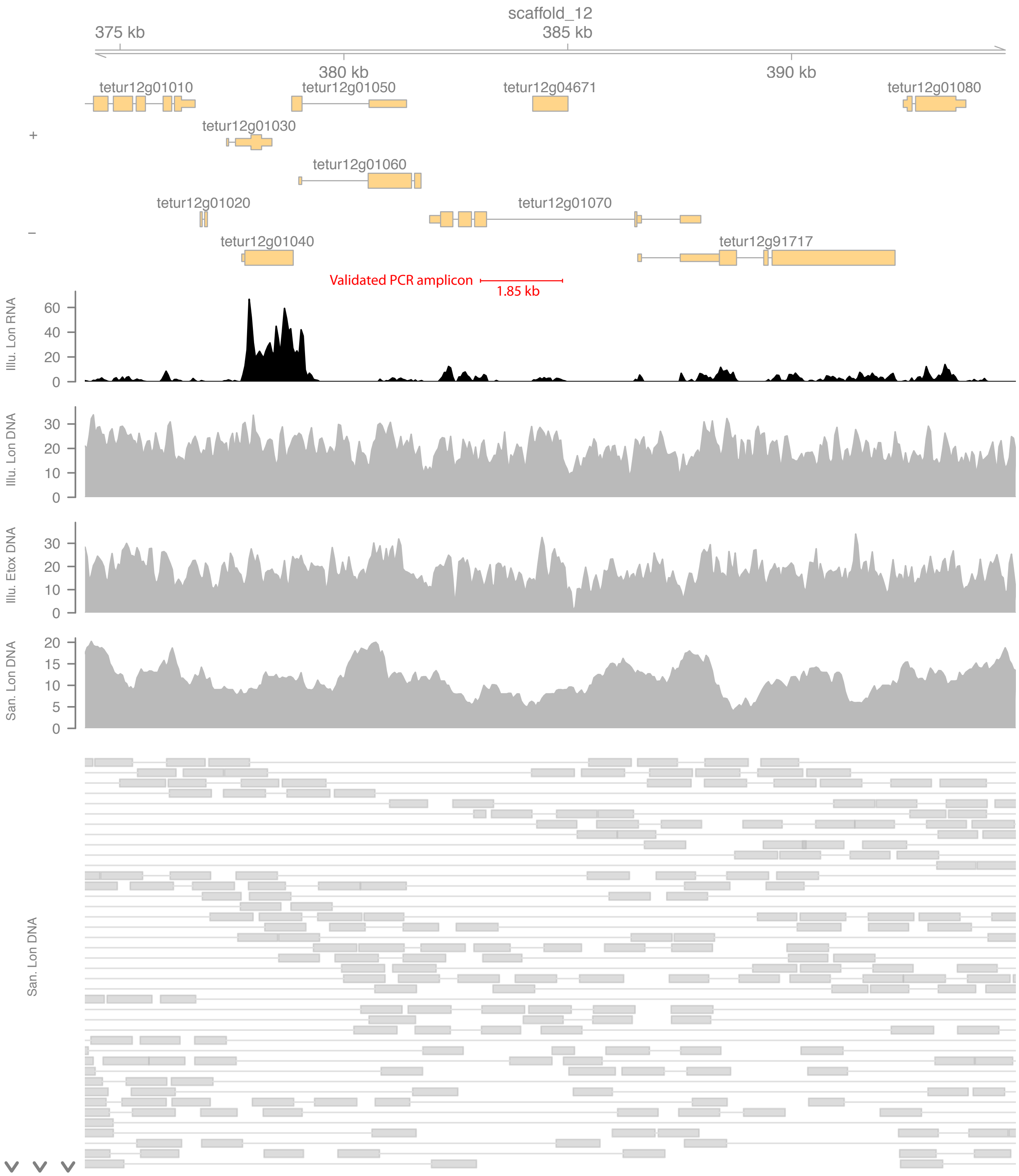

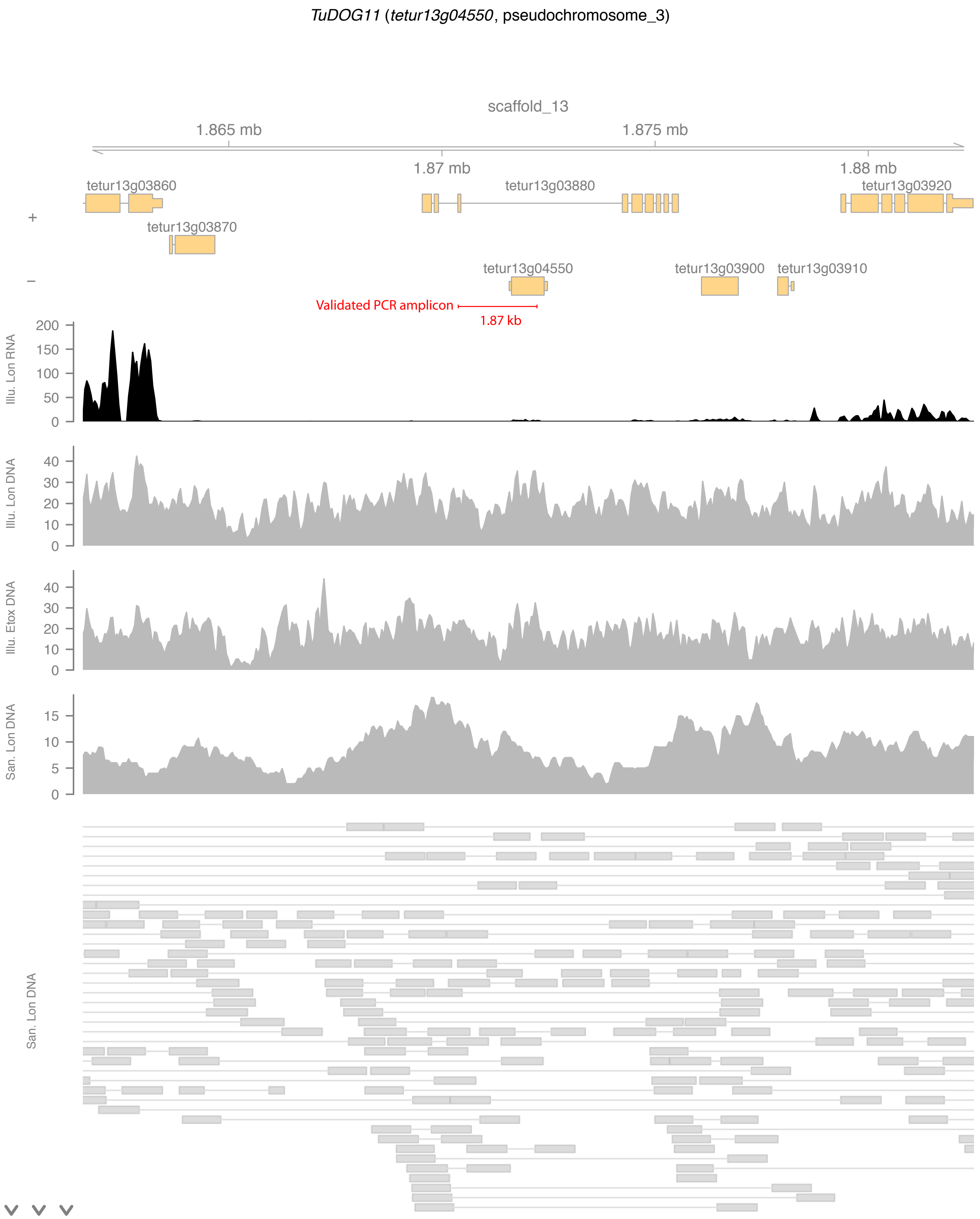

TuDOG11 (tetur13g04550, pseudochromosome_3)
scaffold_13
1.865 mb
1.875 mb
1.87 mb
1.88 mb
tetur13g03860
tetur13g03880
tetur13g03920
+
tetur13g03870
–
tetur13g04550
tetur13g03900
tetur13g03910
Validated PCR amplicon
1.87 kb
Illu. Lon RNA
Illu. Lon DNA
Illu. Etox DNA
San. Lon DNA
San. Lon DNA

TuDOG12 (*tetur19g02300*, pseudochromosome_2)

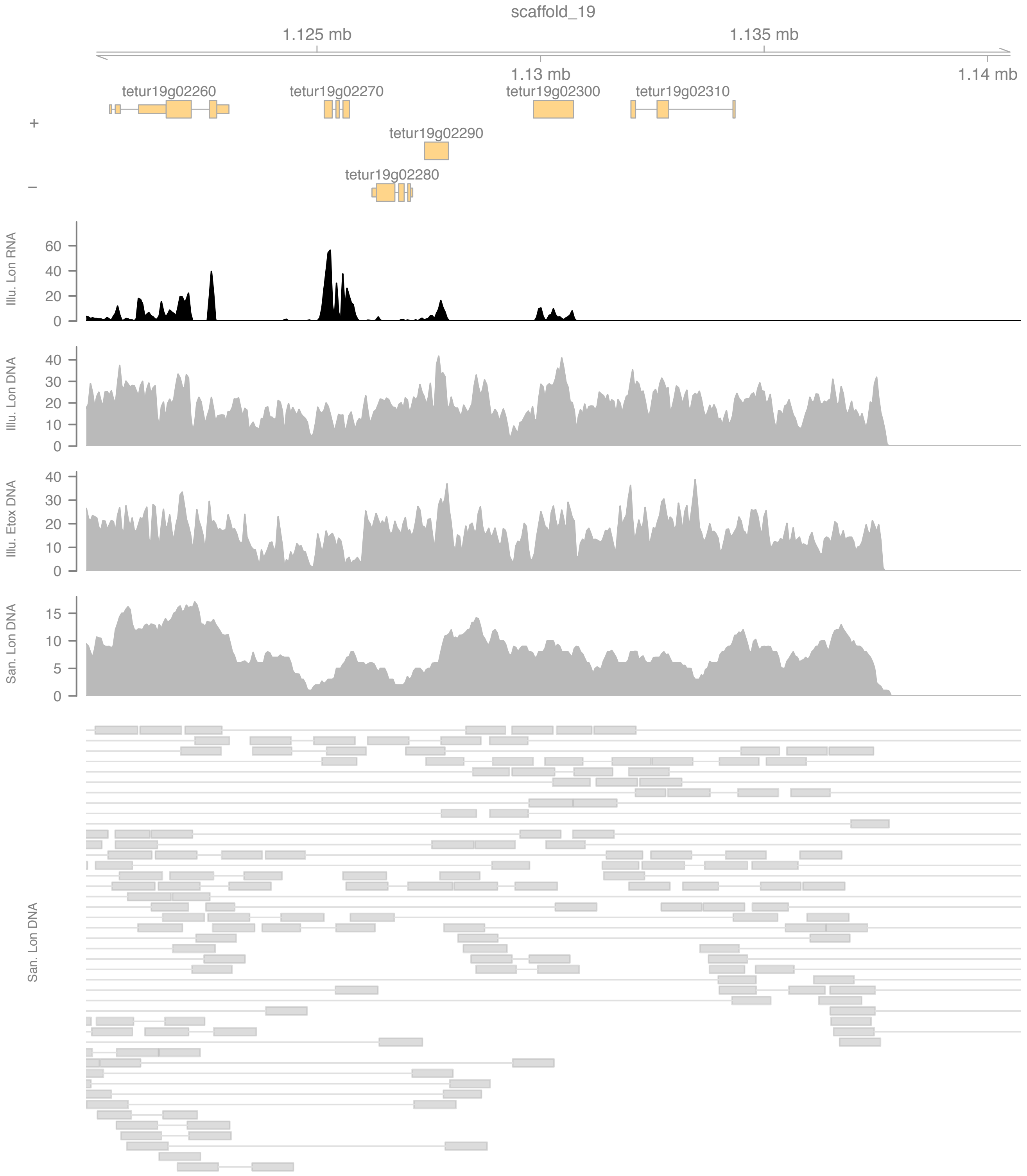

TuDOG13 (*tetur19g03360*, pseudochromosome_2)

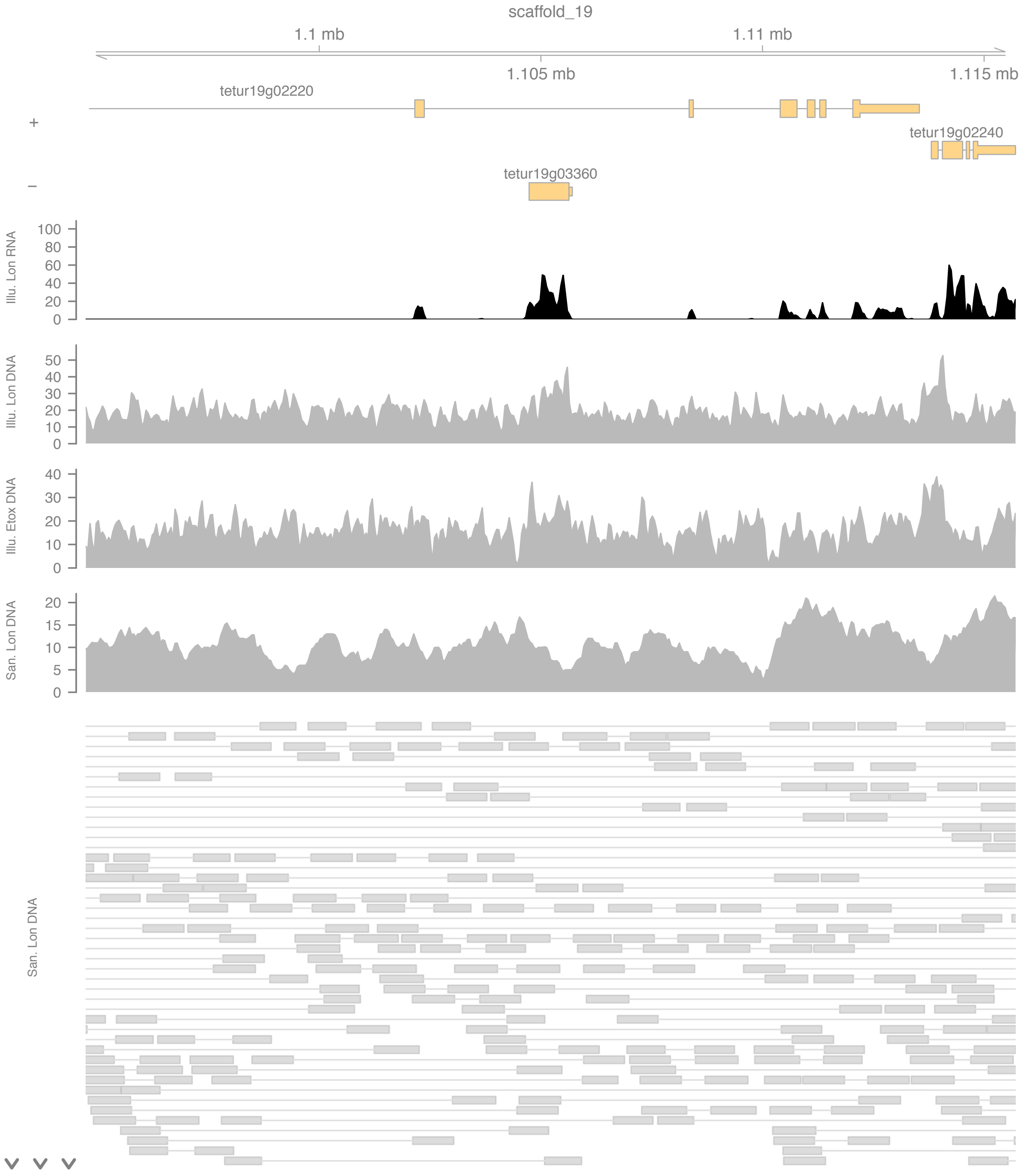

TuDOG14 (*tetur20g01160*, pseudochromosome_3)

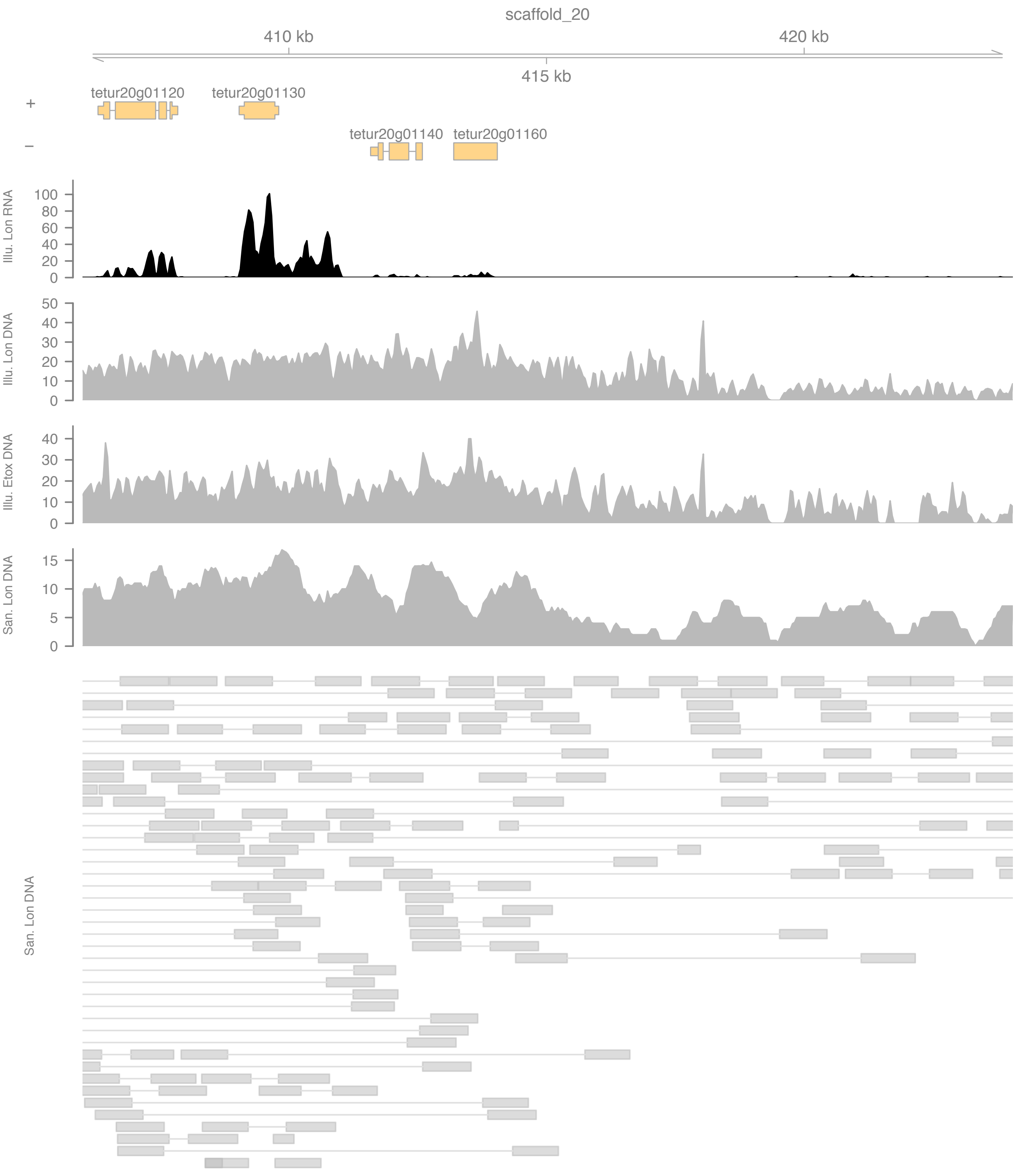

TuDOG15 (*tetur20g01790*, pseudochromosome_3)

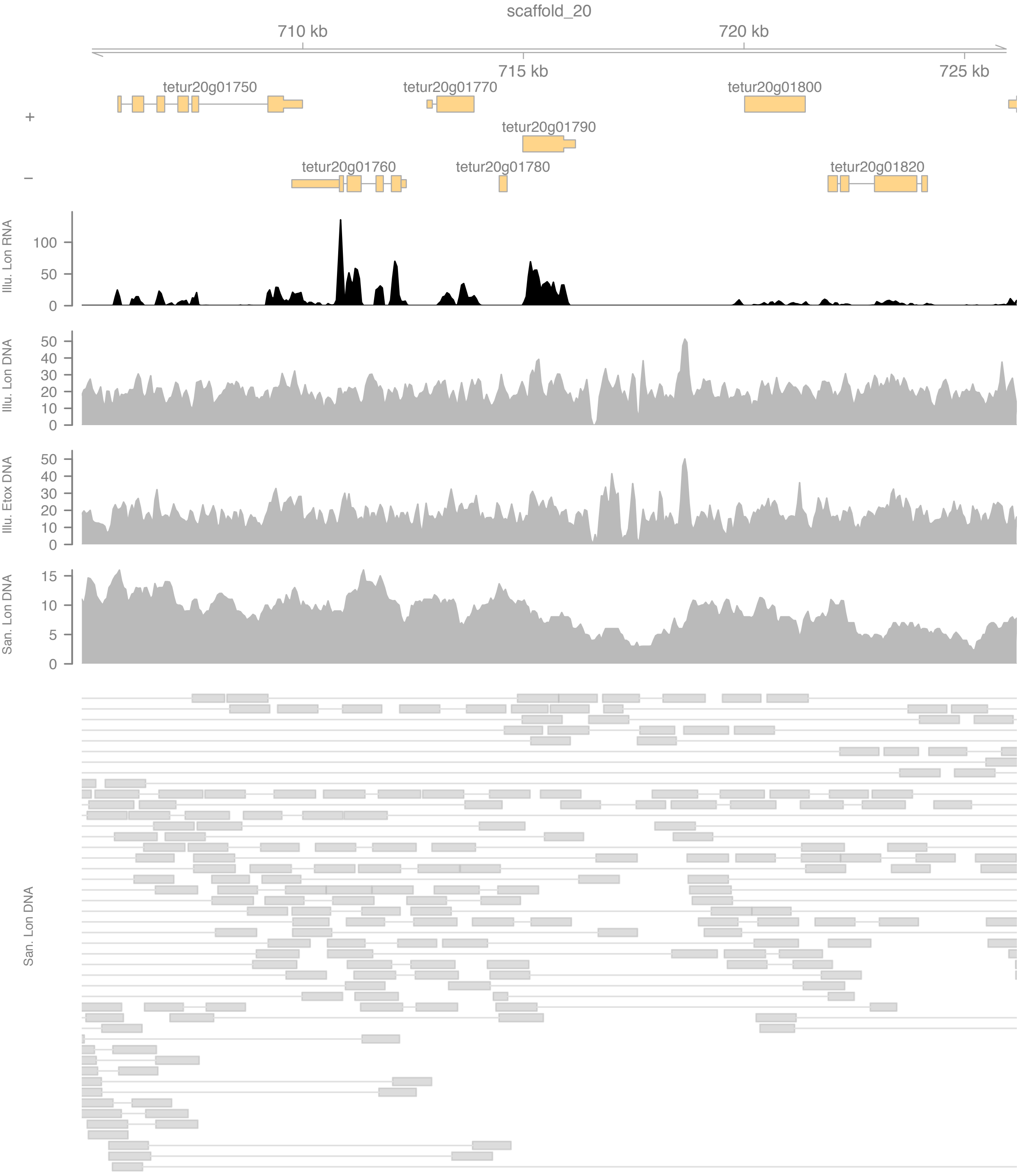

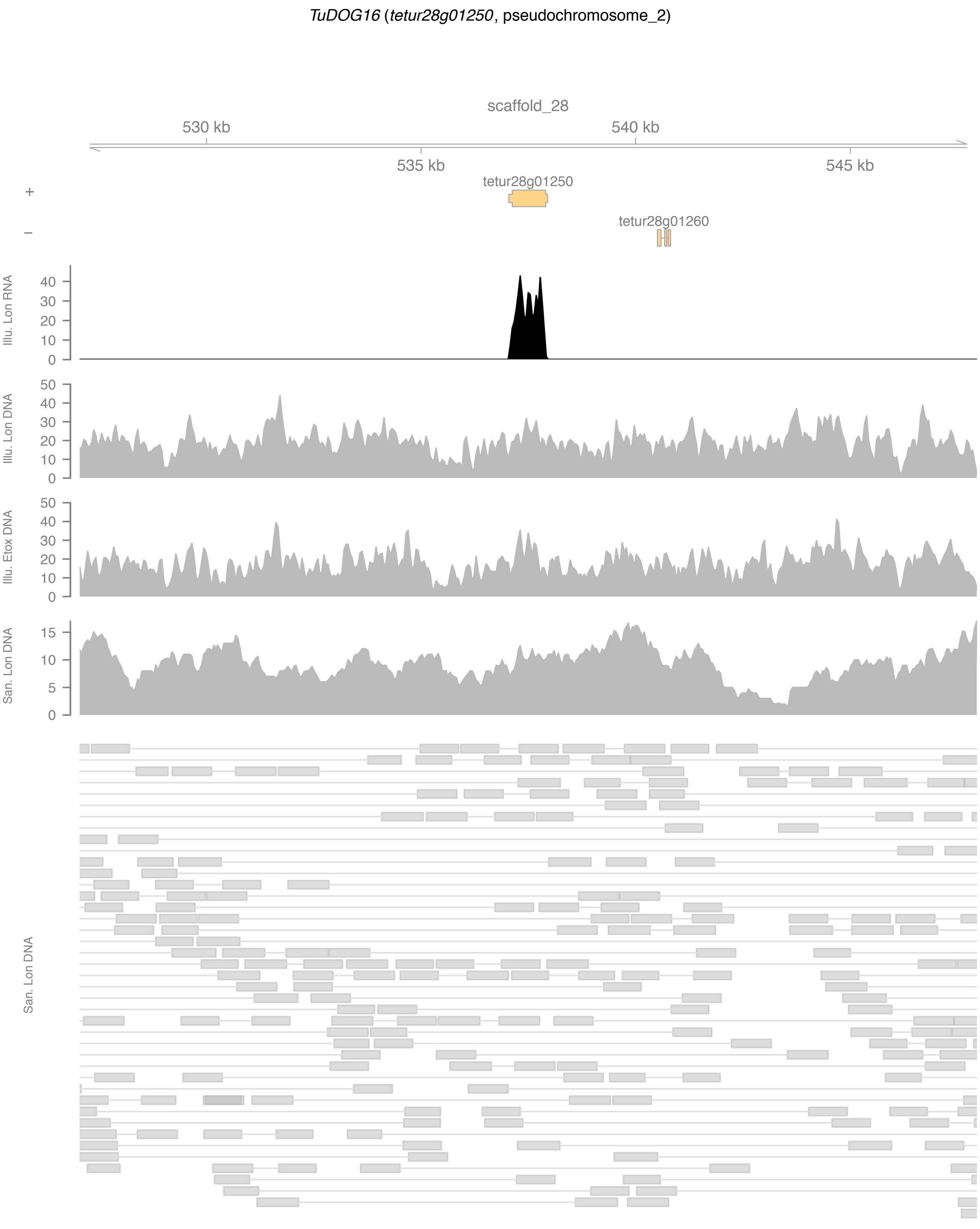

TuDOG16 (tetur28g01250, pseudochromosome_2)
scaffold_28
530 kb
540 kb
535 kb
545 kb
+
–
tetur28g01250
tetur28g01260
Illu. Lon RNA
Illu. Lon DNA
Illu. Etox DNA
San. Lon DNA

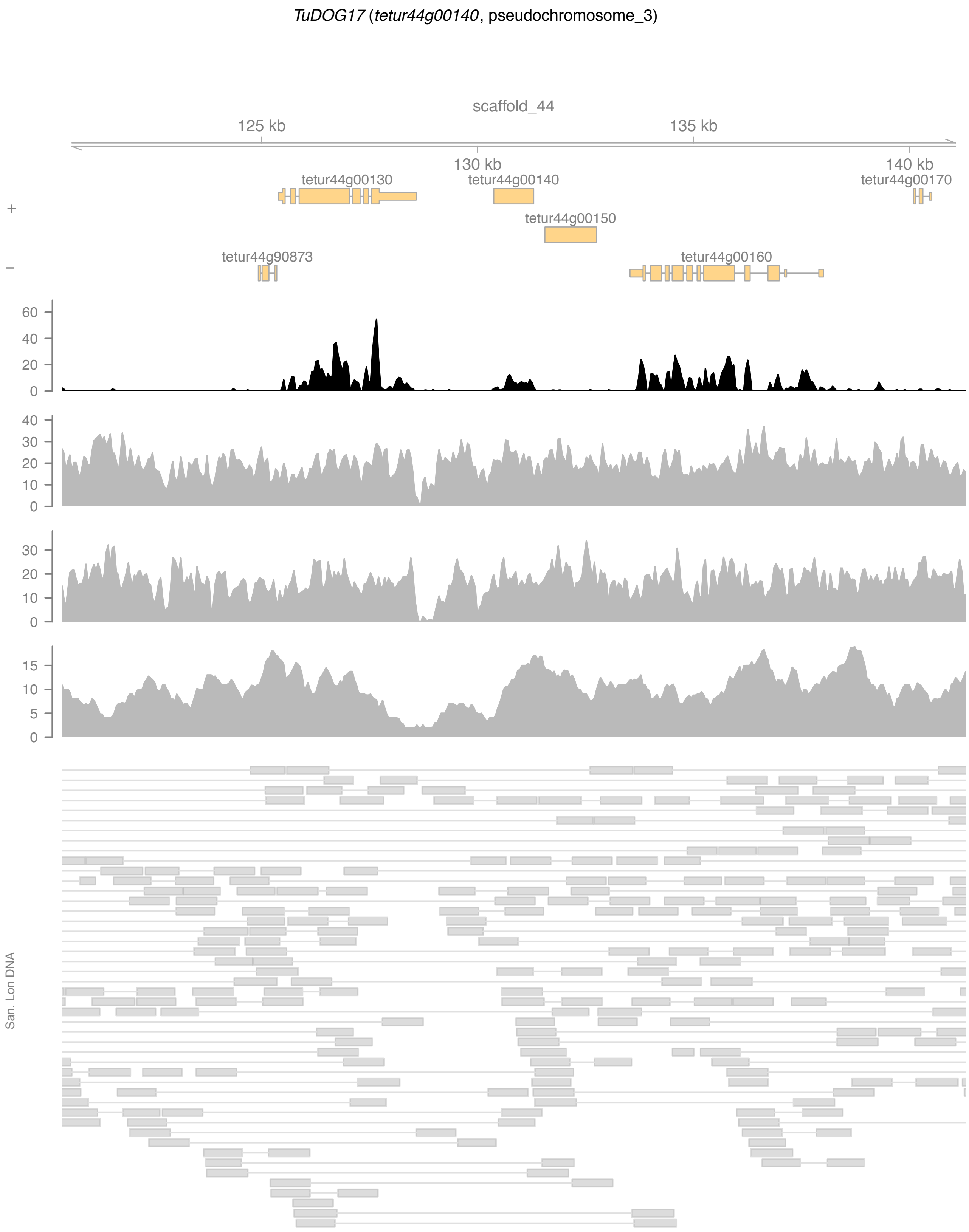

TuDOG17 (tetur44g00140, pseudochromosome_3)
scaffold_44
125 kb
130 kb
135 kb
140 kb
tetur44g00130
tetur44g00140
tetur44g00150
tetur44g00170
tetur44g90873
tetur44g00160
+
–
Illu. Lon RNA
Illu. Lon DNA
Illu. Etox DNA
San. Lon DNA
San. Lon DNA
