# Supplementary figures and images for: Intradiol ring cleavage dioxygenases from herbivorous spider mites as a new detoxification enzyme family in animals
Source: BMC Biol. 2022 Jun 4;20:131. doi: 10.1186/s12915-022-01323-1 (PMC9167512; doi:10.1186/s12915-022-01323-1)

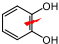

Catechol

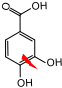

Protocatechuic acid

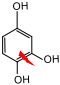

Hydroxyquinol

Supplement: Supplementary file 16 — Additional file 16: Figure S1. Chemical structures of the three products formed from funneling reactions utilized by microorganisms in the breakdown of organic compounds. Catechol and protocatechuic acid degradation proceeds via the β-ketoadipate pathway while hydroxyquinol is formed from protocatechuate and degraded in an alternative pathway found in some bacteria and fungi. [file 12915_2022_1323_MOESM16_ESM.pdf]

A

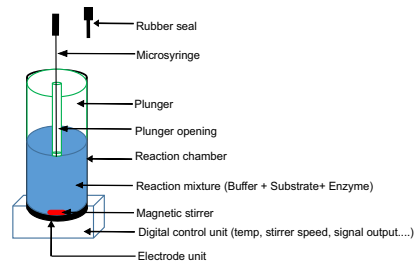

B

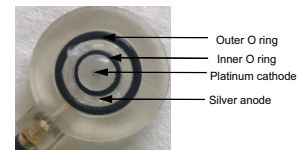

C

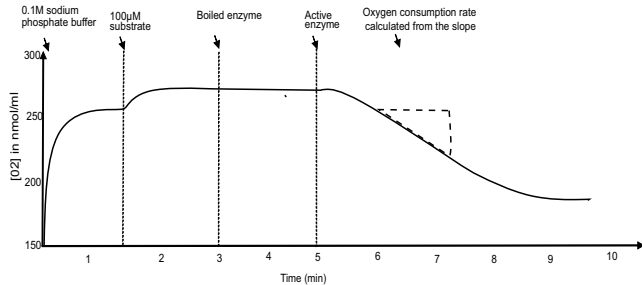

D

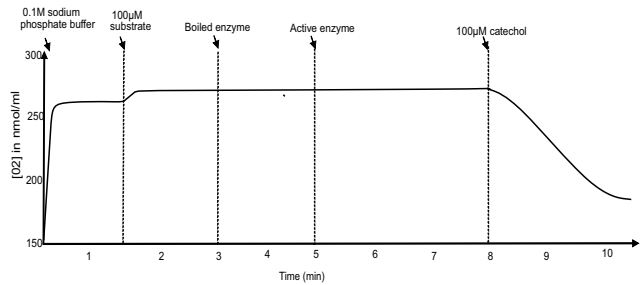

Supplement: Supplementary file 17 — Additional file 17: Figure S2. Schematic of oxygen consumption assay with Clark-type electrode. (A) The Clark type electrode reaction chamber. The rubber seal replaces the microsyringe after injection of the various components into the system. It serves to seal the chamber from external oxygen. (B) A zoom into the electrode unit. The platinum cathode is surrounded by a well that serves as a reservoir for electrolyte solution (50% potassium chloride). During the reaction, the electrolyte is ionized and initiates a current flow from anode to cathode. The current is equivalent to the oxygen concentration in the media. (C) Typical oxygen consumption curve observed when there is substrate cleavage. (D) The curve observed in the absence of substrate cleavage. In the absence of substrate cleavage, catechol was added at the end of the reaction as a control for activity. [file 12915_2022_1323_MOESM17_ESM.pdf]

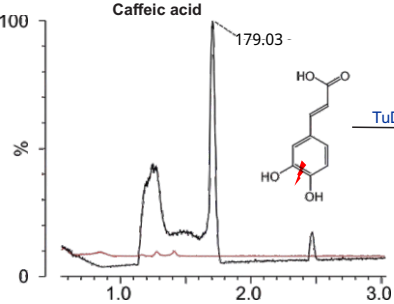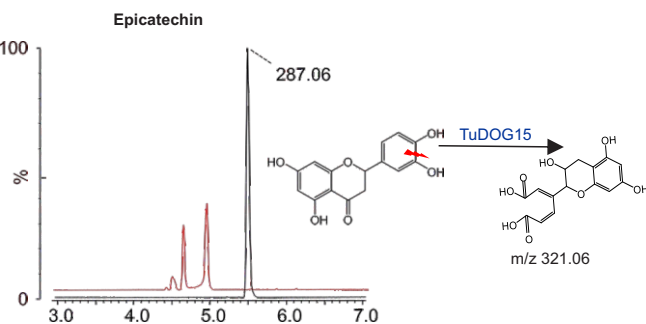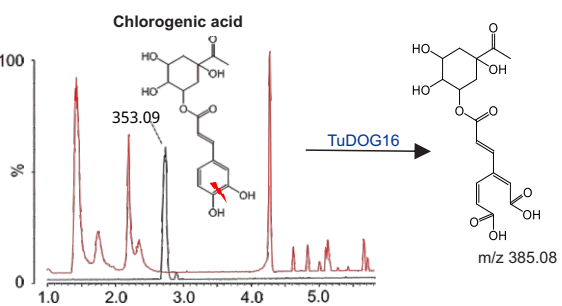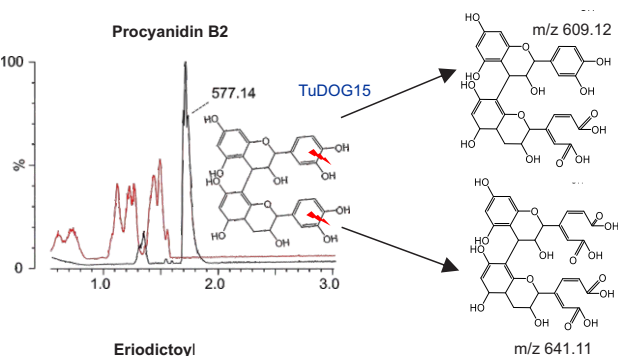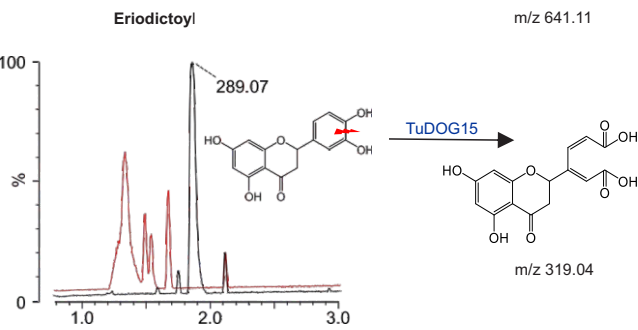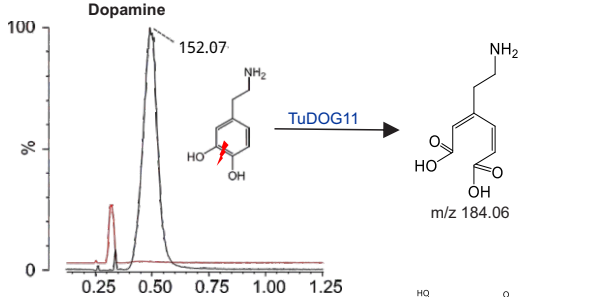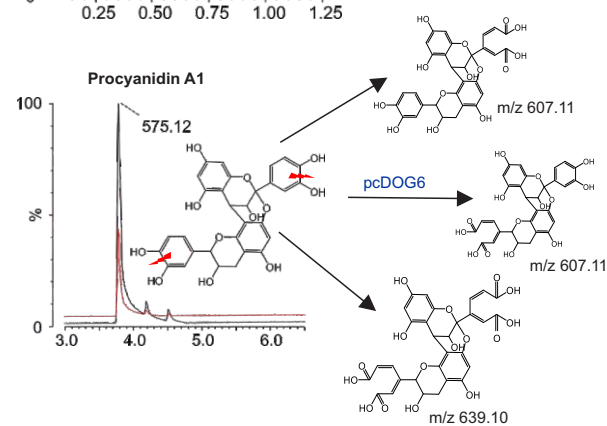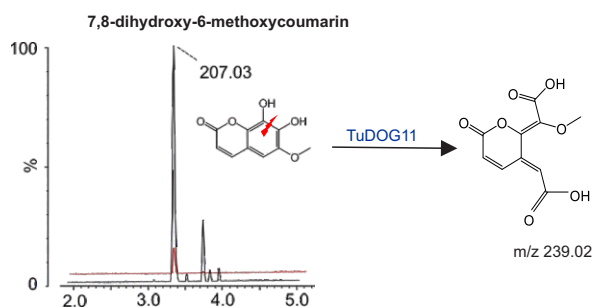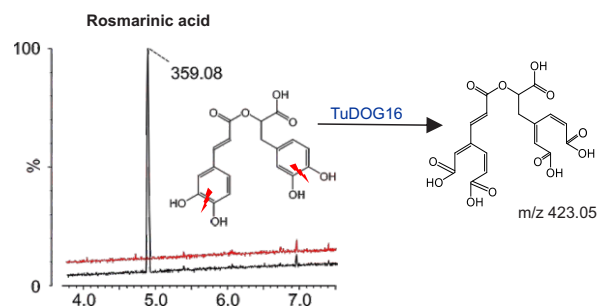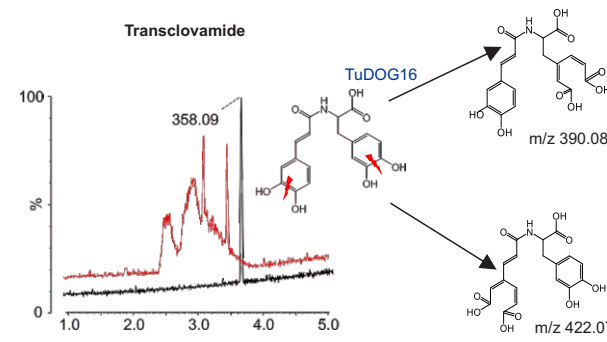

Supplement: Supplementary file 18 — Additional file 18: Figure S3. Spectra of selected substrates and their metabolites as detected in negative mode by UPLC-MS. Black peaks represent the substrate while red peaks depict the metabolites. Structures of both the substrate and the metabolite are as shown with their mass to charge ratios. The DOG used in the assay is indicated in blue. A thunderbolt sign shows the ortho-cleavage position. [file 12915_2022_1323_MOESM18_ESM.pdf]

TuDOG11

antisense probe

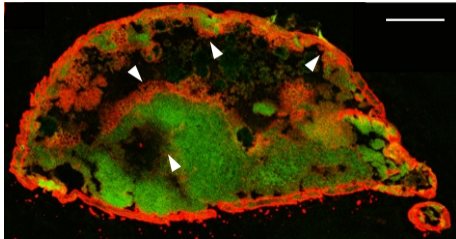

TuDOG3

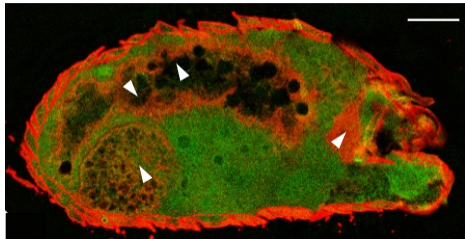

sense probe(control)

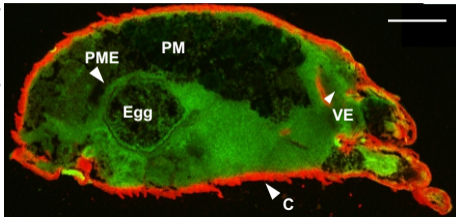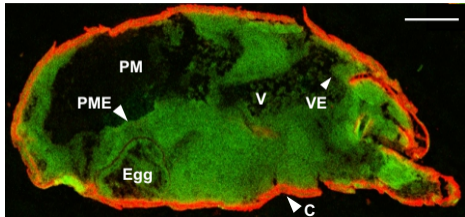

Supplement: Supplementary file 20 — Additional file 20: Figure S5.in situ localization of T. urticae DOG genes (TuDOG11 and TuDOG3). A DIG-labelled antisense probe was used for hybridization and the signal was developed using anti-DIG-AP and FastRed as substrate. The reaction product is visible as a red signal (the cuticle got red staining in both antisense and sense treatment so we consider that as false signals) while the spider mite body shows green auto fluorescence. Abbreviations: C, cuticle; PME, posterior midgut epithelium; PM, posterior midgut; V, ventriculus; VE, ventricular epithelium; Scale bars: 100 μm. [file 12915_2022_1323_MOESM20_ESM.pdf]

A

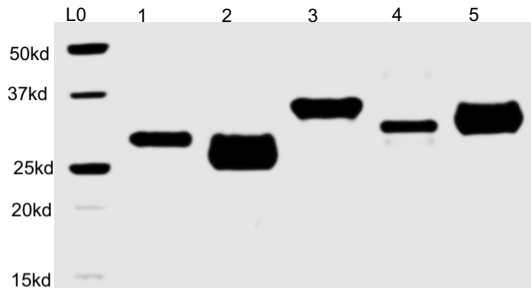

L0- precision plus protein standards (All blue)

1- TuDOG1

2- TuDOG7

3- TuDOG11

4- TuDOG15

5- TuDOG16

B

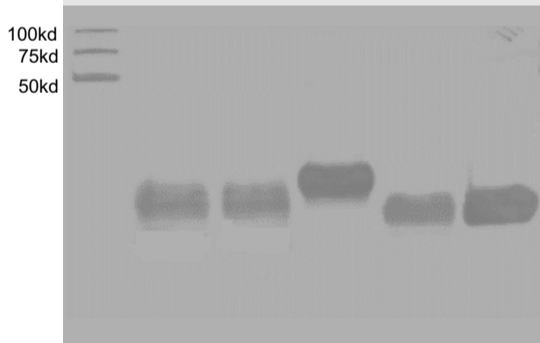

Supplement: Supplementary file 21 — Additional file 21: Figure S6. Stain-free SDS-PAGE (panel a) and western blot (panel b) of purified recombinant T. urticae DOGs. The 6x His tagged proteins are between 25-37 kDa. [file 12915_2022_1323_MOESM21_ESM.pdf]
