# Supplementary material for: Intradiol ring cleavage dioxygenases from herbivorous spider mites as a new detoxification enzyme family in animals
Source: BMC Biol. 2022 Jun 4;20:131. doi: 10.1186/s12915-022-01323-1 (PMC9167512; doi:10.1186/s12915-022-01323-1)
Supplement: Supplementary file 19 — Additional file 19: Figure S4. PCR amplicons of TuDOG7, TuDOG10 and TuDOG11 and their neighboring intron containing eukaryotic genes. [file 12915_2022_1323_MOESM19_ESM.pdf]

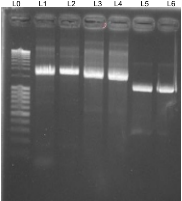

L0-Mass ruler mix DNA ladder

L1 &L2- *TuDOG11* and eukaryotic SSUH2 homolog isoform X2 (*tetur13g03880*). 1.87kb amplicon

L3 &L4- *TuDOG10* and eukaryotic glutaminyl peptide cyclotransferase (*tetur12g01070)*. 1.85kb amplicon

L5 &L6- *TuDOG7* and eukaryotic guanylate kinase (*tetur07g05920*). 1.23kb amplicon
